# A platform for parallel TCR cloning and testing enables anti-neoantigen tumor immunotherapy

**DOI:** 10.1172/jci.insight.203622

**Published:** 2026-04-28

**Authors:** Alexander M. Rowe, Smriti Chaurasia, Wenzhong Wei, Laura García-Diéguez, Katherine Tempro, Johnathon G. Schiebel, Christy Smolak, Alexander Muralles, Daniel Wikenheiser, Kevin Quann, Collin Pirner, Kentin Codispot, Mark J. Shlomchik, Warren D. Shlomchik

**Affiliations:** 1 Department of Immunology, University of Pittsburgh, Pittsburgh, Pennsylvania, USA.; 2 Department of Medicine, Division of Oncology and Malignant Hematology, Pittsburgh, Pennsylvania, USA.; 3 Bluesphere Bio, Pittsburgh, Pennsylvania, USA.; 4 Starzl Transplant Institute, Pittsburgh, Pennsylvania, USA.

**Keywords:** Immunology, Oncology, Cancer immunotherapy, Immunotherapy, T cell receptor

## Abstract

Tumor-infiltrating CD8 cells recognize neoantigens created by tumor-specific mutations. Nonetheless, even after checkpoint inhibitor therapy, most patients’ tumors progress. A deeper understanding of antitumor responses could facilitate development of better therapies. To enable such studies, we applied TCXpress, a high throughput platform that clones fully expressible TCRs from single cells into retroviral or lentiviral vectors without sequencing or gene synthesis, to study TCRs from CD8 cells infiltrating mouse MC38 tumors. We expressed cloned TCRs in reporter cells and interrogated TCR specificity by coculturing them with B6WT3 cells transduced with tandem minigenes encoding predicted neoantigens. We isolated TCRs reactive against epitopes from mutant *Rpl18*, *Adpgk*, *Psmd2*, and *Zc3h7b* along with self-reactive TCRs that recognized normal B6 and MC38 cells. Importantly, we successfully treated MC38-bearing mice with T cells transduced with anti-Rpl18 TCRs. These results establish a system that could be used to study many types of T cell responses and validate a therapeutic approach that could be tested in the clinic.

## Abstract 2

Cancers express abnormal proteins and can be recognized by immune cells. We isolated anti-tumor receptors from these cells and used them to treat tumors.

## Graphical abstract

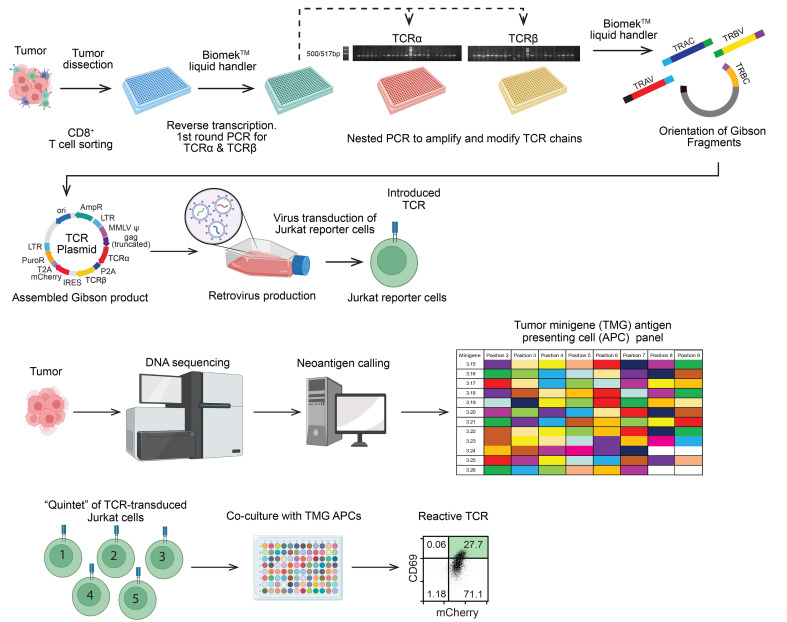

## Introduction

During the last several decades, there has been an explosion in the understanding of naturally occurring and augmented T cell immunity against cancer (1–3). This knowledge underpins the remarkable success of reagents that inhibit CTLA4 and PD-1 inhibitory functions, which in turn enhance antitumor T cell responses (4, 5). Both natural and provoked antitumor T cell responses can target neoantigens, which include variant epitopes created by tumor-specific mutations (2, 6–17). Commensurately, tumors with the highest mutation burden are the most responsive to checkpoint inhibitors (2, 7, 9, 18).

Despite the success of checkpoint inhibition, many patients never respond, and most responding patients eventually relapse. Mechanisms underlying these failures are incompletely understood, but likely include lack of a sufficient tumor-responsive TCR repertoire, immune selection for tumors with mutations that diminish tumor recognition by T cells, and a “suppressive” tumor microenvironment that impedes T cell entry or function (19–28). Elucidating mechanisms of checkpoint inhibitor failure would be enabled by better methods to clone tumor-reactive TCRs and match them to their specificities (3, 29).

The ability to clone anti-neoantigen TCRs also unlocks the possibility of creating therapeutic T cell products that express them. Such products could rescue patients for whom conventional checkpoint inhibition has failed. This potential has been demonstrated by the success of tumor-infiltrating lymphocyte (TIL) therapies, even in patients in whom checkpoint inhibitors failed (30). T cell products with defined specificities created by TCR transduction could be more efficacious than TIL products, which require prolonged culture, and would be more amenable to genome editing designed to overcome resistance mechanisms.

With this background in mind, we have developed methodologies to clone paired TCRα and TCRβ chains from single CD8^+^ T cells and match them to their neoantigen targets. We engineered our system such that many TCRs could be functionally analyzed at a reasonable cost and in reasonable time, as desired TCRs could be rare. By allowing many TCRs to be economically screened, we need not restrict the library to TCRs of predicted specificities (for example by cloning TCRs from cells that bind MHCI-peptide multimers; ref. 31) or to those that come from expanded clones, as could be ascertained by single-cell RNA-Seq (scRNA-Seq) (32). This approach also works around pipelines that require nucleic acid sequencing followed by gene synthesis of these TCRs, as the costs of synthesizing hundreds of TCRs, then cloning them prior to testing them, could be prohibitive (32).

Here, we report the development of a process (TCXpress) that clones TCRα and TCRβ variable regions amplified from single cells directly into retroviral backbones without nucleic acid sequencing or gene synthesis, enabling their rapid expression and functional testing in a parallel fashion. Cloned TCRs were expressed in reporter cell lines that sensitively read out TCR crosslinking. These reporters were cocultured with artificial antigen presenting cells (APCs) transduced with retrovirus that express tandem neoantigen minigene (TMG) constructs for presentation as MHCI-associated peptide neoantigens. We applied this system to clone TCRs from CD8 cells infiltrating implanted MC38 tumor cells. We identified TCRs recognizing multiple neoantigens, and unexpectedly, discovered that TILs include CD8 cells expressing autoreactive TCRs. Finally, we created T cell products expressing anti-neoantigen TCRs that successfully treated MC38 tumors, thus providing proof of principle for an approach that could be applied in the clinic.

## Results

### Cloning TCRs from TILs.

**Figure 1:**
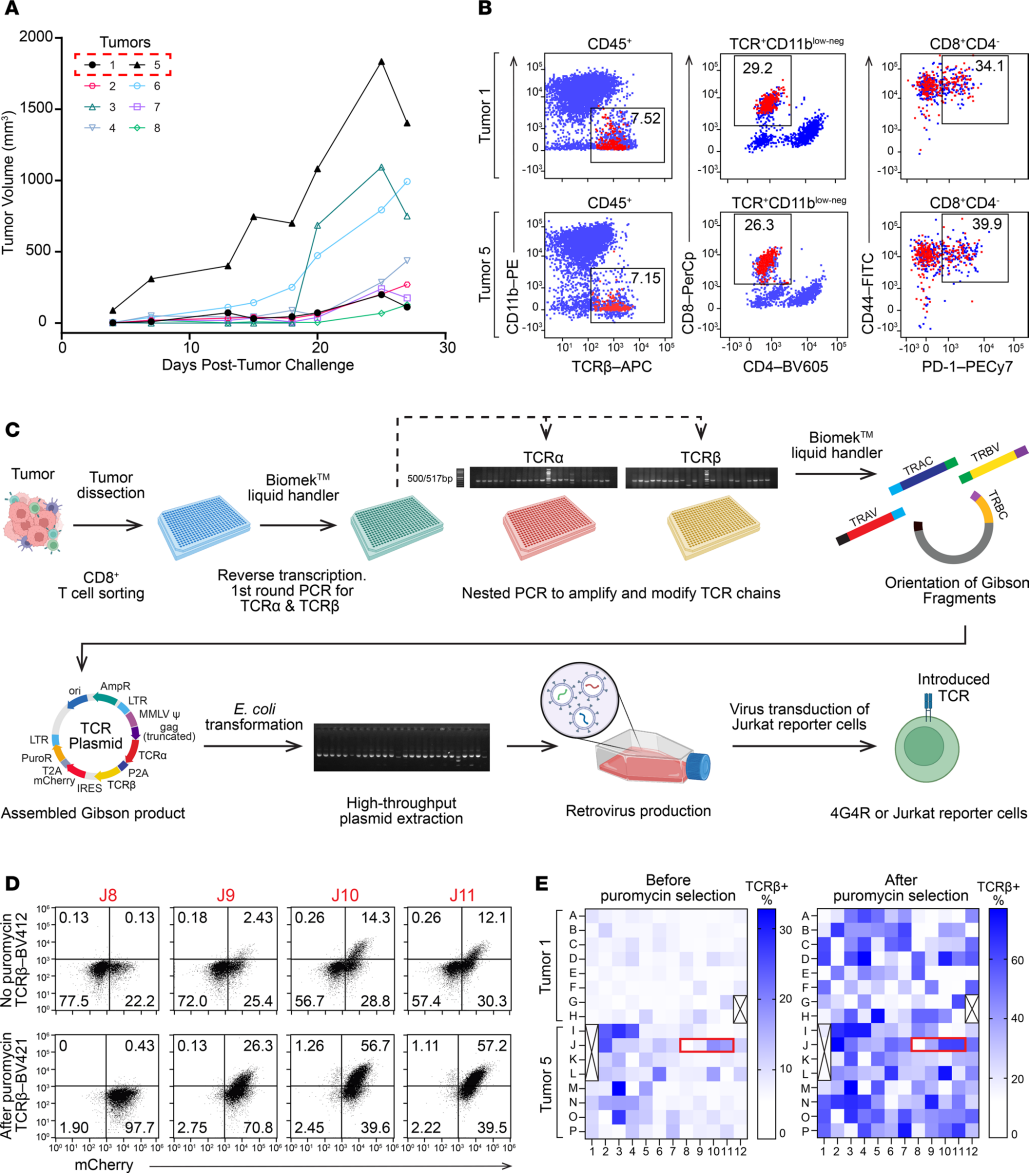
MC38 tumor establishment and cloning of TCRs from single cell–sorted tumor-infiltrating CD8 cells. (**A**) B6 mice were injected intradermally with 5 × 10^5^ MC38 cells and monitored for tumor growth. (**B**) Four weeks later, tumors from mouse 1 and 5 were harvested and digested and CD45^+^CD8^+^ CD44^+^TCRβ^+^CD11b^–^CD4^–^ cells were single cell–sorted for cloning. Sorted cells are depicted in red; percentages in gates are from an analytic sample collected just prior to sorting. PD-1 expression was captured but was not a sort-parameter. The TCXpress process is shown in (**C**). cDNA is generated within each well followed by first round amplification of the TCRα and TCRβ variable regions. The product is split, and TCRα and TCRβ chains are separately amplified, simultaneously adding ends for Gibson cloning. These are assembled with a TCRα constant region (TRAC) fragment and an MSCV-based vector that includes the TCRβ constant region (TRBC). Compatible Gibson cloning ends are color coded. *E*. *coli* are transformed with the Gibson product, followed by plasmid and retrovirus generation. (**D**) TCRβ and mCherry expression of Jurkat cell lines transduced with TCRs J8, J9, J10, and J11 before and after puromycin selection. J8 did not express a functional TCR despite puromycin enriching for mCherry^+^ cells. (**E**) Heatmaps representing percentages of TCRβ^+^ cells for each TCR-transduced Jurkat culture, before and after puromycin selection. Flow cytometry data from Jurkat lines in the red boxes are shown in **D**.

C57Bl/6 (B6) mice were injected intradermally with MC38-Leiden cells (hereafter MC38 cells) and followed for tumor growth (Figure 1A). We chose to study the response against MC38 cells given its in vivo immunogenicity, which suggests a high tumor mutation burden. From tumors 1 and 5, we single cell–sorted 320 tumor-infiltrating CD45^+^CD8^+^TCRβ^+^CD11b^–^CD4^–^ cells into 384-well plates; approximately a third of these cells were PD-1^hi^ (Figure 1B). TCRα and TCRβ variable regions were amplified from single cells (See Methods, Supplemental Methods, and Figure 1C) employing single-cell cDNA synthesis and a nested PCR-based TCR amplification strategy using a custom primer set (Supplemental Table 1) that adds ends for Gibson cloning. These amplified products were purified and then Gibson assembled with 2 other DNA molecules: (a) a fragment encoding the TCRα constant region; and (b) a retroviral backbone that includes the TCRβ constant region. Both the amplified TCRα and TCRβ variable regions were cloned in frame to the constant regions, thereby creating an expressible TCR (Figure 1C). Next, 94 and 92 purified TCRα/TCRβ pairs (Supplemental Figure 1, A–D) from tumors 1 and 5 (respectively), were Gibson assembled into the retroviral backbone, which also encodes mCherry and a puromycin resistance gene (puroR; Figure 1C). Each Gibson product was transfected into bacteria, and plasmid was purified from these bulk cultures without the prior isolation of individual colonies. VSV-G–pseudotyped retrovirus was generated from the resultant plasmid DNA, followed by transduction of Jurkat reporter cells modified to express human CD8α with CRISPR/Cas9 inactivation of TCRα and TCRβ genes. Mouse TCRs function in human T cells (including Jurkat cells), and human CD8α recognizes mouse MHCI molecules (33–36).

Transduced Jurkat lines were puromycin-selected, which increased the percentages of mCherry^+^TCRβ^+^ cells, yielding 145 TCRβ^+^ lines (78% of input Gibson reactions; Figure 1, D and E). That there were lines with very low or no detectable TCR expression despite their being mCherry^+^ may have been due to infidelity in TCR amplification and/or the Gibson assembly.

### Creating APCs expressing putative neoantigens.

To identify potential MC38-encoded neoantigens, we compared whole-genome sequencing data of our MC38 cells with the reference B6 genome GRCm38. We used published MC38 RNA-Seq data (37) to identify putative neoantigens derived from expressed genes. From these data, we generated a list of neoantigens created by nonsynonymous mutations, deletions, or frameshift mutations (Supplemental Table 2).

**Figure 2:**
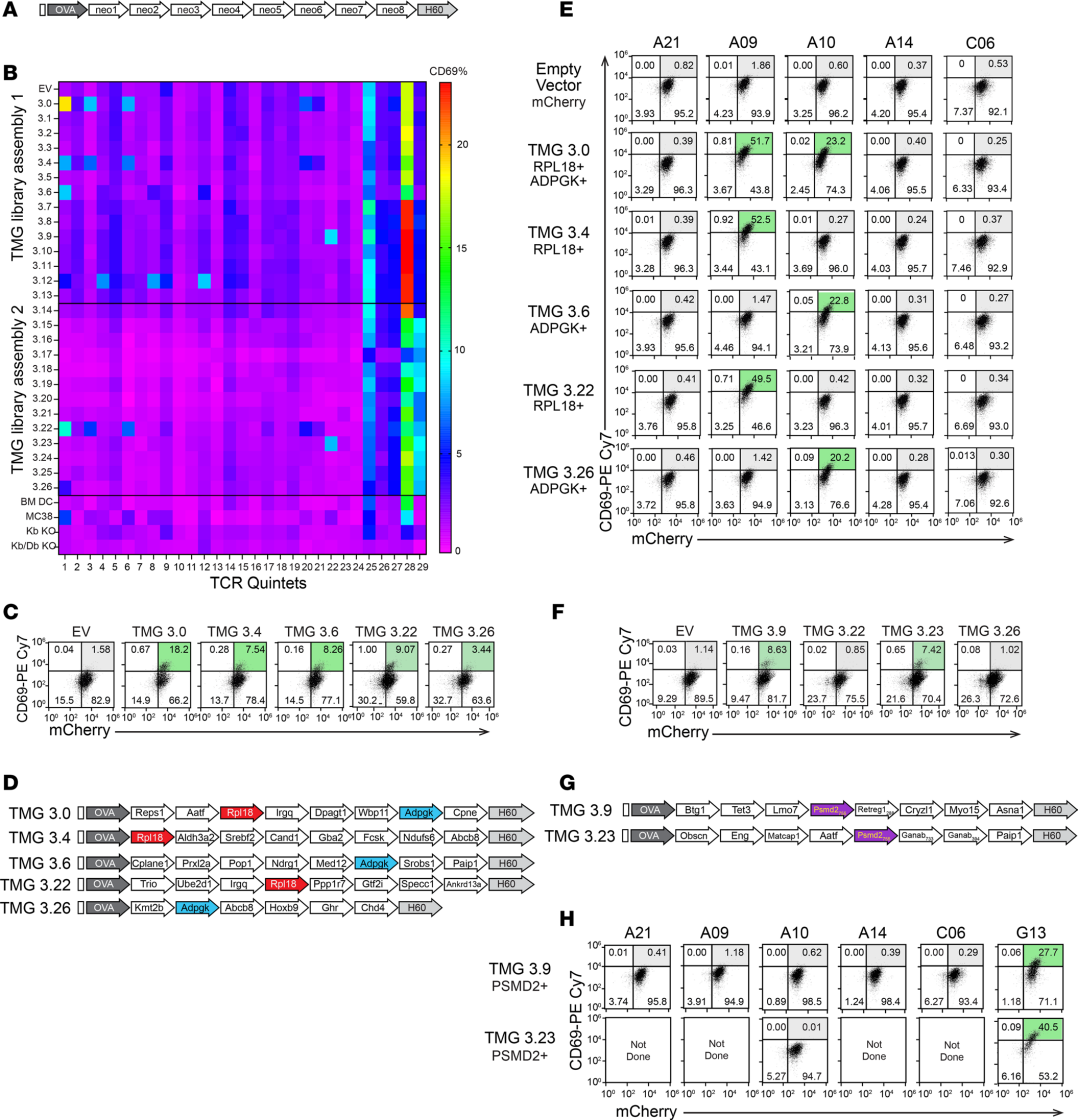
Identification of neoantigen-reactive TCRs. (**A**) TMGs encoding putative neoantigens flanked by epitopes from OVA (SIINFEKL) and H60 (LTFNYRNL) were cloned into retroviral vectors; see also Supplemental Figure 2A. (**B**) Pools of 5 Jurkat lines (quintets), each expressing a TCR cloned from a single cell, were cocultured with B6WT3 cells transduced with TMG retroviruses. The percentage of CD69^+^ cells in each culture was determined by flow cytometry and plotted as a heatmap. (**C**) Representative flow plots from quintet 1 showing reactivities against B6WT3 cells expressing TMGs 3.0, 3.4, 3.6, 3.22, 3.36 or an empty vector (EV) negative control. (**D**) Putative neoantigens encoded by TMGs 3.0, 3.4, 3.5, 3.6, 3.22, and 3.26. The individual resource lines from quintet 1 were separately cocultured with TMGs 3.0, 3.4, 3.6, 3.22, and 3.26. (**E**) TCR A09 reacted with TMGs 3.0, 3.4, and 3.22, which shared expression of Rpl18, whereas TCR A10 reacted with TMGs 3.0, 3.6, and 3.26, which shared expression of ADPGK. (**F** and **G**) Quintet 22 reacted with TMGs 3.9 and 3.23, which only shared expression of PSMD2. (**H**) The single lines from quintet 22 were individually cultured with TMG-APCs 3.9 and 3.23, revealing TCR G13 to be reactive against PSMD2.

To assess whether putative neoantigens were targets of cloned TCRs, we cocultured TCR-expressing Jurkat cells with B6WT3 APCs engineered to stably express TMGs encoding up to 8 putative neoantigens. Within the TMGs, each putative neoantigen-containing peptide was represented by at least 25 aa, with the predicted MHCI-binding peptide in the center (Supplemental Table 2). Each TMG sequence was flanked by K^b^-restricted epitopes from OVA (SIINFEKL) on the amino terminus and the minor histocompatibility antigen H60 (LTFNYRNL) on the carboxy terminus (Figure 2A) (38, 39). The inclusion of these defined antigens allowed us to use reporter cells expressing OVA or H60-reactive TCRs as positive controls for minigene epitope expression. Further, we could screen for construct expression using the 25D1 antibody, which recognizes K^b^-SIINFEKL complexes (40). These TMG constructs were Gibson-cloned into a retroviral vector similar to that used to express TCRs (Supplemental Figure 2A) and retrovirus was generated. B6WT3 cells were infected with single TMG-encoding retroviruses and puromycin-selected, which led to clear 25D1 staining (Supplemental Figure 2B). In this fashion, a library of TMG-expressing B6WT3 sublines was produced to represent all of the neoantigen targets.

We created 26 B6WT3 lines expressing 100 putative neoantigens. Each neoantigen was expressed in at least 2 different constructs (assemblies 1 and 2; Supplemental Figure 2C) to facilitate the decoding of TCR reactivities from a single screen. TMG construct 3.0 encoded 8 putative neoantigens identified by others, which were also identified by our analyses (37, 41). Prior to coculturing TMG-encoding APC lines with TCR-transduced Jurkat lines, they were treated with IFN-γ to enhance their APC function, which predictably led to upregulation of MHCI (Supplemental Figure 2D).

We combined individual TCR-expressing Jurkat lines into groups of 5 (quintets). Each quintet was cocultured with each TMG-expressing B6WT3 line and then assessed for CD69 upregulation. As negative controls, each quintet was cocultured with B6WT3 cells infected with an empty vector (EV) and B6WT3 cells that were CRISPR-edited to lack K^b^ or K^b^ and D^b^. Quintets were also cocultured with B6 BM-derived DCs and IFN-γ–treated MC38 cells to test for autoreactivity or tumor recognition, respectively.

We observed 2 broad types of reactivity (Figure 2B). Some quintets reacted against specific TMGs (e.g., quintets 1, 21, and 22), whereas others reacted against most TMGs (quintets 28 and 29) and also against MC38 cells and DCs. It was expected that the percentages of CD69^+^ cells in “positive” quintets would be low because typically only 1 of 5 lines would be reactive. Furthermore, fewer than 100% of cells from each line would be expected to express the correct TCR due to expression of the wrong TCRα chain, potential nucleic acid amplification errors, and their derivation from plasmid generated from the entire Gibson cloning reaction, without the selection of colonies encoding a single correctly assembled TCRα/TCRβ pair.

Quintet 1 reacted against TMGs 3.0, 3.4, 3.6, 3.22, and 3.26 (Figure 2C). That TMG 3.0 induced the highest percentage of CD69^+^ cells among quintet 1 suggested that quintet 1 might contain 2 positive TCRs. Inspection of the neoantigen identities (Figure 2D) revealed that Rpl18 was expressed in TMGs 3.0, 3.4, and 3.22, while ADPGK was expressed by TMGs 3.0, 3.6, and 3.26, suggesting that quintet 1 had TCRs reactive against mutant Rpl18 and ADPGK (Rpl18^mut^; ADPGK^mut^).

To determine which TCRs within quintet 1 were reactive and against which neoantigens, we cultured each single Jurkat line with each TMG APC (Figure 2E). TCR A09 reacted against TMGs 3.0, 3.4, and 3.22, which only shared Rpl18^mut^. TCR A10 reacted against TMGs 3.0, 3.6, and 3.26, which only had ADPGK^mut^ in common. Therefore, quintet 1 had 2 anti-neoantigen TCRs, one each reactive against Rpl18^mut^ or ADPGK^mut^. Using this same approach, we identified additional Rpl18^mut^-reactive TCRs from quintets 3, 6, 20, and 21 (Table 1).

Quintet 22 reacted against TMGs 3.9 and 3.23 (Figure 2F), which shared a mutant PSMD2 (Figure 2G); one-to-one cultures mapped reactivity to clone G13 (Figure 2H). Several quintets reacted against TMG 3.12. However, they did not react against TMGs in assembly 2 that expressed the same putative neoantigens; this reactivity was not pursued further.

Rpl18^mut^ and ADPGK^mut^ have been described as targets for anti-MC38 CD8 responses (37, 41) and our cloning of multiple anti-Rpl18^mut^ TCRs confirms this. To focus on other neoantigens, we performed a second TCR cloning campaign aiming to exclude TCRs reactive against Rpl18^mut^ or ADPGK^mut^. MC38 cells were implanted (Supplemental Figure 3A) and TILs were isolated. In an attempt to omit Rpl18^mut^- and ADPGK^mut^-reactive cells, we stained cells with MHCI-dextramers against Rpl18^mut^ (dex^Rpl18^) and ADPGK^mut^ (dex^ADPGK^). There was a clear dex^Rpl18+^ but not a dex^ADPGK+^ population. We thus single cell–sorted 352 dex^Rpl18–^ cells with low dex^ADPGK^ staining (Supplemental Figure 3B) and cloned TCRs as in Figure 1. In this campaign, we sequenced TCRβ chains prior to choosing which TCRs to advance to Gibson cloning, enabling us to prioritize TCRs isolated more than once and to limit repeated screening of multiple isolates of expanded clones. We cloned 187 TCRs into retroviral vectors with 184 of the TCR-transduced Jurkat lines expressing TCRβ. Next, 150 transduced Jurkat lines with the best TCRβ expression were screened against the TMG APC library (Supplemental Figure 3C). Of these, 28 TCRs were from clones captured more than once (Supplemental Figure 3D).

In addition to screening these new TCRs against our original TMG library, we returned to the genome sequencing data and selected an additional 112 putative neoantigens for screening (Supplemental Table 2, Supplemental Figure 2, and Supplemental Figure 3E). We incorporated these into 2 assemblies totaling 28 TMG constructs, with each neoantigen represented in a different context in each assembly (TMGs 4.1 to 4.14 and TMGs 4.15 to 4.28). Thirty quintets from the second TCR library were cocultured with APCs expressing TMGs 4.1 to 4.14 and APCs expressing TMGs 3.0 to 3.13 from the first screen (Supplemental Figure 3F).

TMGs 3.0 and 3.4 were reactive against quintets 4, 9, 12, 13, and 14, which only shared Rpl18^mut^. By reacting the individual Jurkat lines against TMG APCs 3.0 and 3.4, we identified 7 unique anti-Rpl18^mut^ TCRs (Table 1). That these were isolated from dex^Rpl18–^ cells may reflect that TCRs were downregulated sufficiently prior to staining to diminish dextramer binding.

TMG 4.8 reacted against quintets 3, 7, 8, 9, 10, 19, 25, and 30. We cocultured each of the individual Jurkat lines from these quintets with TMG 4.8 APCs; the positive lines were cocultured with the second APC assembly (TMGs 4.15 to 4.28). At least 1 individual culture from each of these quintets was reactive only against TMGs 4.8 and 4.23, which share an epitope derived from the zinc finger protein Zc3h7b. For example, quintet 19 reacted against TMG 4.8 (Supplemental Figure 3G). From quintet 19, only clone J18 reacted against TMGs 4.8 and 4.23 (Supplemental Figure 3, I and J). In total, 10 TCRs reactive against Zc3h7b^mut^ were isolated, of which 9 were unique (Table 1). From these quintets, we also identified additional anti-Rpl18^mut^ TCRs (Table 1). By screening TCRs from our first campaign against TMG library 4, we found 1 additional anti-Zc3h7b^mut^ TCR (Table 1).

### Molecular deconvolution.

As a consequence of our Gibson-cloning process, the resultant plasmid DNA contained a mix of molecular species that could include more than 1 TCRα chain along with errors acquired during amplification and cloning. To isolate a single molecular TCRα/TCRβ pair for downstream work, plasmid DNA isolates derived from the Gibson reactions that generated the reactive TCRs were transformed into bacteria and single colonies were used to create retrovirus, followed by transduction of reporter cells.

An example of this process is the deconvolution of the anti-Rpl18 TCR H03. Among the 8 Jurkat lines derived from 8 colony picks, there was surface TCR expression in 7 (Supplemental Figure 4A). Interpretable TCRα and TCRβ sequences were obtained from 6 colonies (Supplemental Figure 4B), all of which used TRAV14-D-1 and TRBV20. However, there were 2 TRAJ-TRAC junctions, reflecting independent TRAV14-D-1 rearrangements, with picks 3, 4, 5, and 7 having one junction and 6 and 8 the other junction. We cocultured Jurkat reporter cells expressing picks 4, 6, 7, and 8 against B6WT3 cells transduced with Rpl18^+^PSMD2^–^ TMG 3.4 and Rpl18^–^PSMD2^+^TMG 3.23. Only cells expressing picks 6 and 8 (Supplemental Figure 4C), sharing the second TCRα rearrangement, supported CD69 upregulation, thereby identifying the correct TCR. We performed a parallel process to deconvolute the anti-PDSM2 TCR G13 (Supplemental Figure 4, D–F). This process of molecular deconvolution was repeated for most Jurkat lines that reacted against 2 TMG APCs that encoded a common putative neoantigen and therefore were likely bona fide.

### Isolation of autoreactive TCRs.

In the first screen, TCR pools 9, 25, 27, 28, and 29 had broad reactivities against all TMG-encoding B6WT3 lines (Figure 2B), B6 BM DCs, and MC38 tumor cells. Importantly, these pools did not react against B6WT3 cells lacking K^b^ and D^b^, indicating MHCI-dependent reactivities. We cocultured each of the single Jurkat lines from these pools against MC38 cells, identifying 7 that were reactive. Molecular deconvolution of these lines isolated a unique autoreactive TCR from each line (Supplemental Figure 5A). Jurkat cells expressing these deconvoluted TCRs were reacted against MC38 cells and WT or K^b^- or D^b^-deficient B6WT3 cells. Clones A16, A22, D17, and I15 were D^b^-restricted; the rest were K^b^-restricted. In the second screen of TCRs from MC38 TIL, quintets 7, 8, 10, 13, 17, 20, and 21 had broad reactivity. From these quintets, 9 individual Jurkat lines responded to both B6WT3 and MC38 cells, demonstrating that autoreactive TCRs were common. We did not pursue these latter TCRs further due to resource restraints.

### Treatment of B16 melanoma with TCR-transduced products.

To facilitate testing of whether T cells transduced with neoantigen-specific TCRs would be effective against MC38 tumors in vivo, we needed to develop methods for creating TCR-redirected products. We sought a well-characterized TCR and a well-characterized in vivo tumor model in order to optimize cell production. Thus, we tested these T cell products in vivo against a B16 melanoma subclone modified to express a TMG encoding epitopes from gp100, H60, TRP1, TRP2, and OVA (B16-TMG; Supplemental Figure 6A). The modified subclone was killed in vitro by activated anti-SIINFEKL OT-1 TCR transgenic T cells (Supplemental Figure 6B) and grew similarly in B6 mice as did parental B16-F10 cells (Supplemental Figure 6C). When implanted, B16-TMG cells generated anti-OVA and anti-H60 CD8 responses (Supplemental Figure 6D). This B16-TMG line was successfully treated in vivo by the adoptive transfer of expanded OT-1 TCR transgenic cells (Supplemental Figure 6E).

To explore optimal expression vectors, we cloned the OT-1 TCRα and TCRβ chains, separated by a T2A sequence, into 2 retroviral backbones. In one, 3′ to the TCR cassette was an IRES-EGFP segment followed by a WPRE sequence; in the second, the IRES-EGFP was deleted but the WPRE sequence was retained (“no-fluoro” vector). To knock down endogenous TCRs, we designed guide RNAs (gRNAs) targeting the constant regions of TCRα and TCRβ; all TCRs studied with transduced products had constant regions modified to be resistant to these gRNAs.

**Figure 3:**
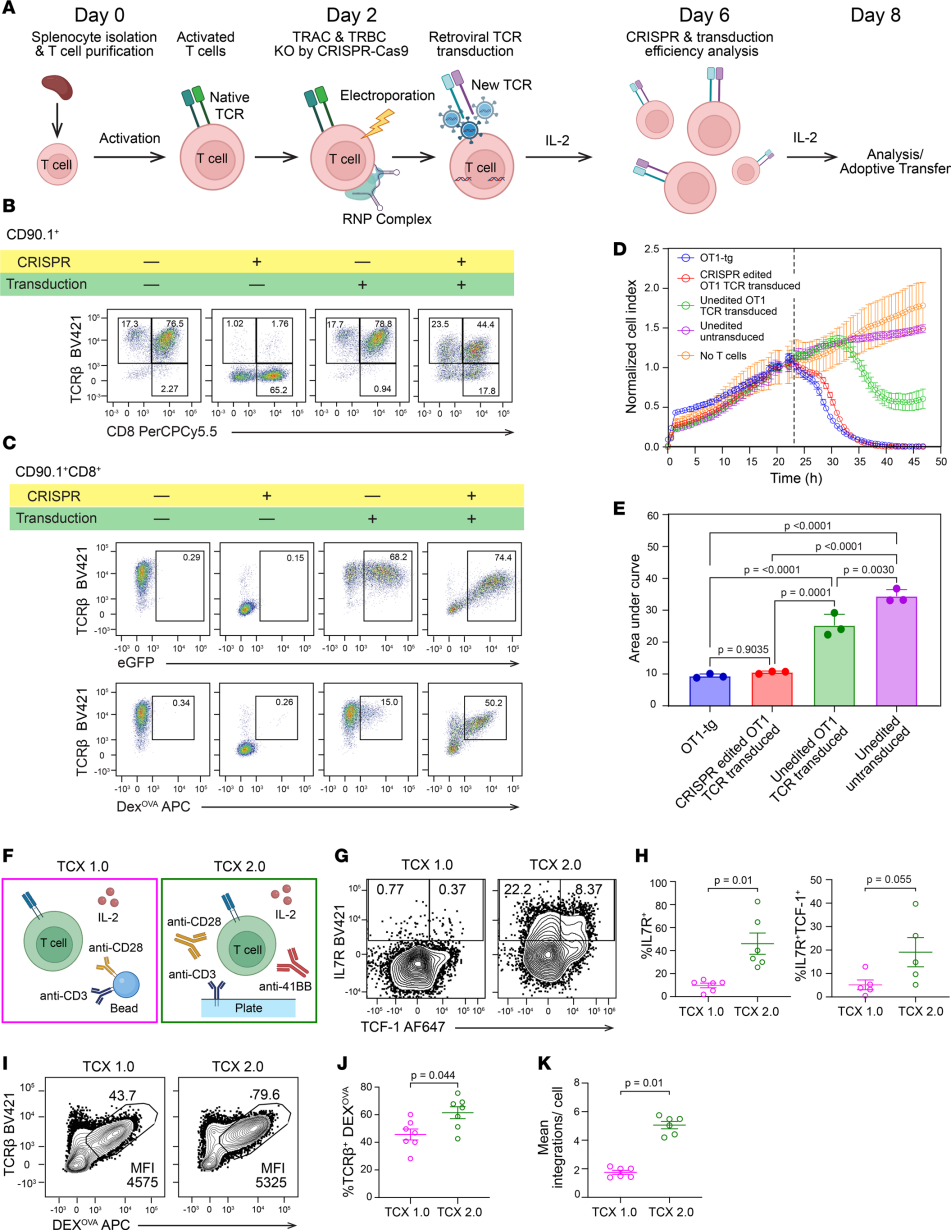
Development of TCX 1.0 and TCX 2.0 T cell products. (**A**) Schematic for TCX 1.0 production. Purified splenic T cells were activated with anti-CD3/CD28 Dynabeads and IL-2 for 2 days. De-beaded cells underwent TCRα/TCRβ chain deletion via electroporation of gRNA/Cas9 complexes. After resting for 1 hour, these cells were transduced with OT-1 TCR-encoding retrovirus (coexpresses GFP) and cultured for 6 additional days in IL-2. (**B** and **C**) Characteristics of cells manufactured as in **A**, with or without CRISPR-TCR deletion or retroviral transduction. (**B**) Staining for TCRβ and CD8 (gated on CD90.1^+^ cells). (**C**) GFP or dex^OVA^ staining versus TCRβ expression (gated on CD90.1^+^CD8^+^ cells). (**D** and **E**) KO of the endogenous TCR dramatically improved dex^OVA^ binding on OT-1 TCR-transduced CD8 cells. The resultant improvement in OT-1 TCR expression translated into better in vitro killing of B16-TMG cells. (**F**) Schematic representation of differences between TCX 1.0 and TCX 2.0 production. (**G** and **H**) T cell activation in TCX 1.0 is with anti-CD3/CD28 beads and IL-2, whereas in TCX 2.0, activation is with plate bound ant-CD3, IL-2, and soluble antibodies against CD28 and 41BB (gated on CD8^+^ cells); a higher percentage of TCX 2.0 CD8 cells were IL-7R^+^ and IL-7R^+^TCF-1^+^. (**I**–**K**) TCX 2.0 led to better dex^OVA^ binding (**I** and **J**) and more viral integrations per cell (**K**). For **G**–**K**, each symbol represents data from a unique T cell product; data were compared using Mann-Whitney rank-sum tests.

In a first approach (TCX 1.0, Figure 3A), T cells were activated with anti-CD3/CD28 Dynabeads in IL-2 for 2 days, followed by electroporation with Cas9/gRNA complexes. Cells were then transduced with OT-1 TCR-encoding retrovirus, followed by culture in IL-2 for 6 days. Carefully titered retrovirus (Supplemental Figure 7) was used to assure consistent MOI across products. TCRα and TCRβ editing was efficient (Figure 3, B and C). When cells were TCR-transduced without CRISPR editing, TCRβ expression was similar to that in cells that underwent neither TCR transduction nor TCR editing (Figure 3B). However, with combined TCR editing and TCR transduction, TCR expression was lower, as most TCR was derived from integrated retrovirus.

Endogenous TCR deletion proved critical for optimal transduced TCR expression. Despite 68% transduction of CD8 cells based on GFP expression, without endogenous TCR editing, dex^OVA^ binding was low (Figure 3C). However, with concomitant TCR KO, dex^OVA^ binding was strong and correlated with overall TCRβ expression (Figure 3C). This improvement in OT-1 TCR expression had functional consequences because TCR-edited and TCR-transduced cells had similar killing as did activated OT-1 TCR transgenic cells, whereas there was less killing when cells were TCR transduced without editing (Figure 3, D and E).

Based on data suggesting that activation of T cells in vitro with anti-41BB agonist antibodies improves their metabolic fitness (42), we incorporated anti-41BB into a second manufacturing approach, wherein we stimulated T cells with plate-bound anti-CD3 and soluble antibodies against CD28 and 41BB, along with IL-2 (TCX 2.0; Figure 3F). The resultant product had a higher frequency of cells expressing IL-7Rα (CD127) and TCF-1 (Figure 3, G and H), which are predictive of better performance (43–47). TCX 2.0 also resulted in improved TCRβ expression and dex^OVA^ binding (Figure 3, I and J). This could have been driven by more retroviral integrations per cell (Figure 3K).

**Figure 4:**
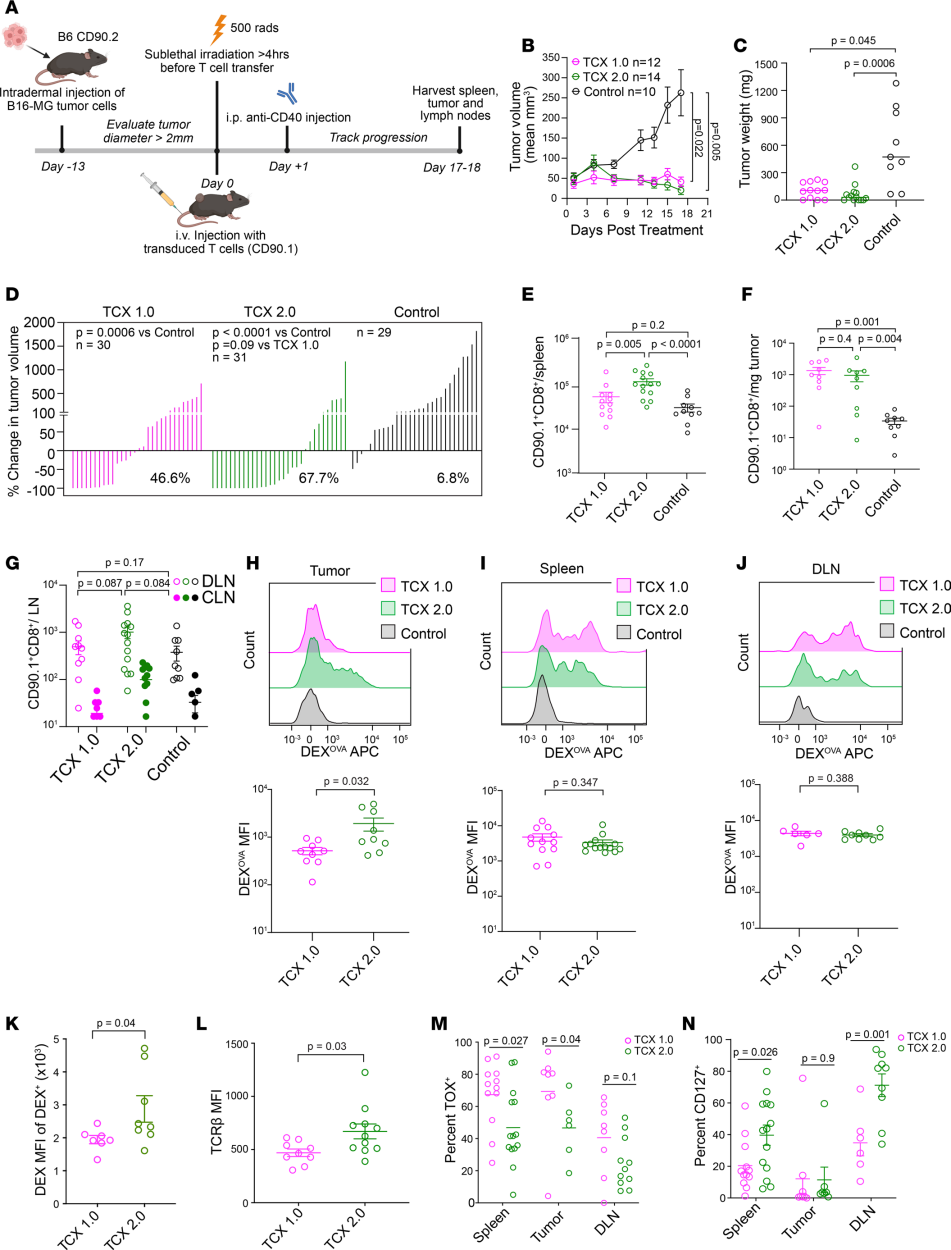
Treatment of B16-TMG melanoma with TCX 1.0 and TCX 2.0 products. (**A**) Treatment schema. B6 CD90.2 mice were injected intradermally with B16-TMG cells on day –13. On day 0, mice with tumors greater than 2 mm received total-body irradiation and CD90.1 TCX 1.0, TCX 2.0, or negative control cells, followed on day 1 with anti-CD40. Tumor size was followed and measured; mice were euthanized by day 18. (**B** and **C**) Both TCX 1.0 and TCX 2.0 cells reduced tumor volume (**B**) and weight (**C**). (**D**) Waterfall plots of change in tumor size from 3 independent experiments combined; mice that died prior to the end of the experiments were censured (see Supplemental Figure 8A). (**E** and **F**) TCX 2.0 CD8 cells were more numerous than TCX 1.0 and control cells in spleen (**E**), whereas there were similar numbers of each recovered from tumor, both greater than control cells (**F**). (**G**) More transferred TCX 1.0, TCX 2.0, and control cells were found in the draining LNs (DLNs) than in nondraining cervical lymph nodes (CLNs). (**H**–**J**) Dex^OVA^ MFIs of TCX 1.0 and 2.0 cells were similar in the spleen and DLNs but were greater in tumor-resident TCX 2.0 cells (top panels representative staining; lower panels MFIs from individual mice). (**K** and **L**) The dex^OVA^ MFIs were higher among dex^OVA+^ TCX intratumor TCX2.0 cells (**K**) perhaps due to higher TCRβ expression (**L**). (**M** and **N**) A greater percentage of TCX1.0 cells were TOX^+^ in spleen and tumor (**M**), whereas a greater fraction of TCX 2.0 cells were CD127^+^ in spleen and DLN (**N**). Data in **B** and **C** are from 1 of 3 experiments with similar results. Differences between groups in **B** were calculated by determining the AUC of tumor volume versus posttreatment day data for each mouse at the end of the experiment and comparing the groups by ANOVA with Tukey’s post hoc test. *P* values in **C** were calculated using a Mann-Whitney rank-sum test. Data in **D**–**N** are combined from 3 independent experiments, and groups were compared using a Fisher’s exact test. Significance of cell numbers or MFIs in **E**–**N** were determined with Mann-Whitney rank-sum tests.

We next tested TCX 1.0 and TCX 2.0 products in T cell immunotherapy of B16-TMG melanoma (Figure 4A). Mice were injected intradermally with B16-TMG cells. When tumors were 2 mm or larger in diameter, mice received total body irradiation (500 cGy) prior to the infusion of 2 × 10^6^ OT-1 TCR-transduced dex^OVA+^ CD8 cells created by TCX 1.0 or TCX 2.0. Though there was variability across experiments, products were 60%–80% CD8^+^ with 40%–70% dex^OVA+^. Control animals received T cells that underwent CRISPR editing without TCR transduction, adjusted to equal the number of cells in the OT-1 products that were not CD8^+^dex^OVA+^. In this way, we compared the efficacy of the TCR-transduced CD8 cells to the contaminating other T cells in the product. The following day, mice were injected i.p. with the agonist anti-CD40 antibody FGK45 as an adjuvant (48, 49), and tumor volumes were followed. Both TCX 1.0 and TCX 2.0 products controlled B16-MG growth (Figure 4, B–D). There was a trend for TCX 2.0 resulting in greater tumor regression relative to TCX 1.0 (Figure 4D; *P* = 0.09).

At euthanization, there were more splenic CD8^+^ TCX 2.0 cells than either control or TCX 1.0 cells (Figure 4E and Supplemental Figure 8B). Intratumoral TCX 2.0 and TCX 1.0 CD8 cells were similar in number and more numerous than control cells (Figure 4F and Supplemental Figure 8C). The numbers of TCX 1.0, TCX 2.0, and control CD8 cells in draining and nondraining cervical lymph nodes (LNs) were also similar, with trends toward more TCX 2.0 cells (Figure 4G). However, the dex^OVA^ MFI of TCX 2.0 CD8 cells was higher than that of TCX 1.0 cells in the tumor but not in the spleen or draining LNs (Figure 4, H–J). Among dex^OVA+^ CD8 cells, the dex^OVA^ MFI was also higher in TCX 2.0 cells (Figure 4K), consistent with a higher TCRβ MFI (Figure 4L). Taken together, these data suggest that in locations bereft of antigen (spleen and LN), TCX 2.0 cells were advantaged, perhaps because a greater fraction expressed CD127 (IL-7Rα) (Figure 4N and Supplemental Figure 8, B and C). Conversely, in the tumor where antigen was available, there could have been selection for cells with higher TCR expression as generated by the TCX 2.0 process (Figure 4J). Compared with TCX2.0 cells, TOX expression was higher among TCX 1.0 cells in tumor and spleen (Figure 4M and Supplemental Figure 8, B and C); this could reflect TCX 1.0 cells having more sustained antigen exposure, consistent with the trend for TCX 1.0 cells less effectively controlling tumor growth (Figure 4D).

### Deeper characterization of anti-neoantigen TCRs.

**Figure 5:**
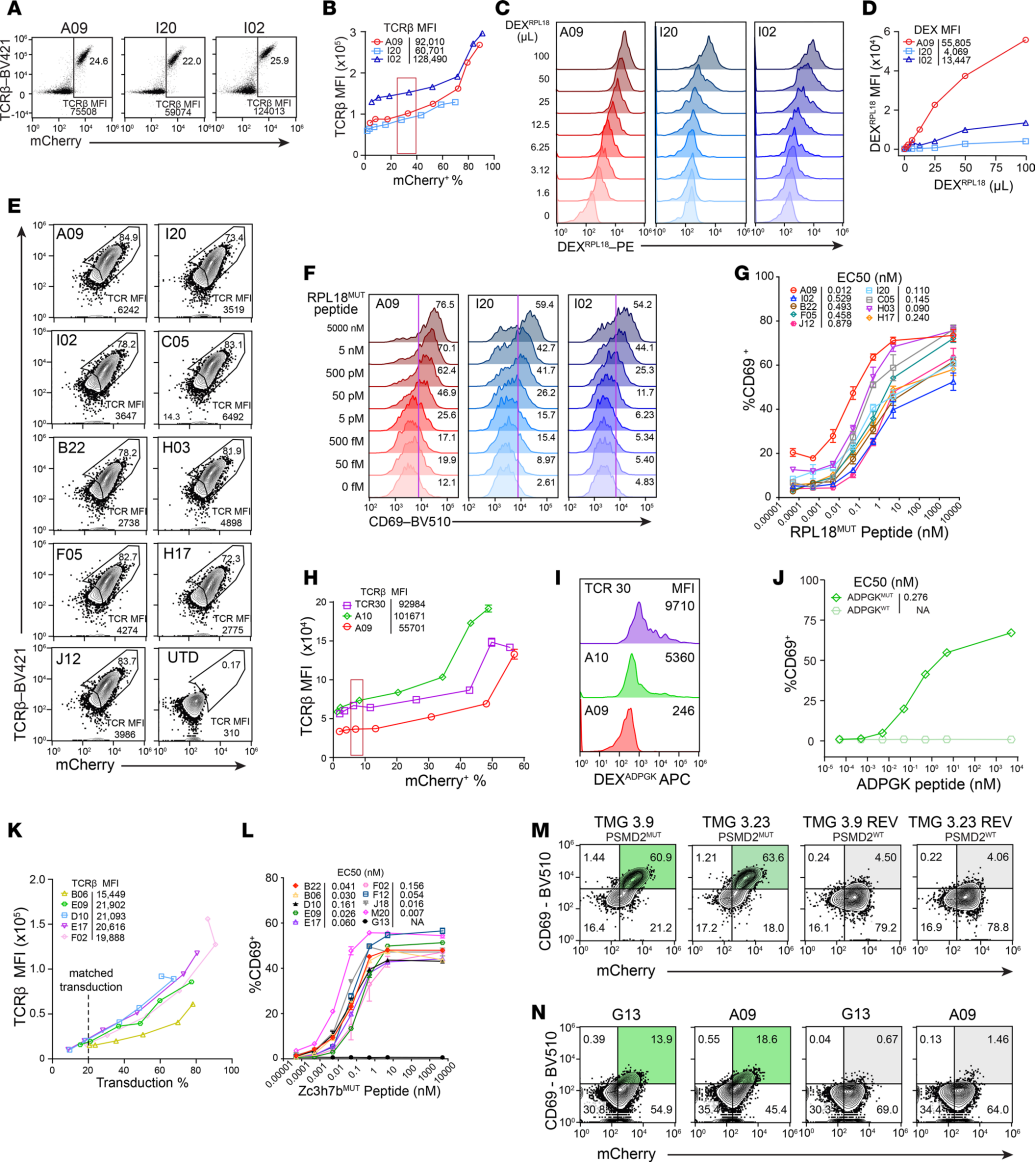
Characterization of anti-neoantigen TCRs. (**A**–**D**) mCherry-expressing retrovirus encoding the anti-Rpl18^mut^ TCRs A09, I20, and I02 were used to infect 4G4R cells over a range of MOIs, and TCRβ expression Rpl18^mut^ -dextramer binding were quantitated. (**A**) Representative flow. (**B**) Plot of percentage of mCherry^+^ versus TCRβ MFI of mCherry^+^ cells; each transduction was done in triplicate. (**C** and **D**) At a matched transduction (see red box in **B**), dex^Rpl18^ binding over a range of concentrations was determined. (**E**) To test functionality, Jurkat reporter cells were transduced with TCRs A09, I20, C05, B22, H03, F05, H17, and J12 and puromycin-selected. (**F** and **G**) These lines were cultured with different concentrations of Rpl18^mut^ peptide-pulsed B6WT3 cells (in triplicate) and the percentages that were CD69^+^ were determined. (**F**) Representative flow for TCRs A09, I20 and I02. (**G**) Peptide concentration/response plots for all TCRs. (**H** and **I**) Retrovirus encoding anti-ADPGK TCRs A10 and TCR30 and control anti-Rpl18 TCR A09 were used in dilution to infect 4G4R cells (in triplicate). Shown are TCRβ expression of mCherry^+^ cells (**H**) and dex^ADPGK^ staining of mCherry^+^ cells at about a 10% transduction (red box in **H** and **I**). (**J**) TCR A10-expressing Jurkat cells were cocultured with mutant or WT ADPGK peptide-pulsed B6WT3 cells (in triplicate) and the percentages of CD69^+^ cells were determined. (**K**) 4G4R cells were retrovirally transduced with anti-Zc3H7b TCRs over a range of MOIs in triplicate, and TCRβ MFIs were determined (of TCRβ^+^ cells). (**L**) Jurkat cells transduced with anti-Zc3H7b TCRs were reacted with B6WT3 cells pulsed with different concentrations of Zc3H7b-mutant peptide, and the percentages of CD69^+^ cells were determined. (**M**) Anti-PSMD2^mut^ G13 TCR-transduced Jurkat cells were cocultured with B6WT3 cells transduced with PSMD2^mut^-encoding TMG 3.9 and 3.23 or versions reverted (REV) to express the WT PSMD2-derived peptide (PSMD2^WT^). (**N**) Anti-PSMD2^mut^ G13 and A09 anti-Rpl18^mut^ TCR-expressing Jurkat cells were reacted against MC38-Leiden cells and MC38-Kerafast cells, which lack the targeted Rpl18 and PSMD2 mutant peptides.

To select TCRs for immunotherapy against MC38 tumors, we characterized several TCRs in depth. TCRs vary in surface expression (50) and affinity for peptide/MHCI complexes. To assess surface expression of Rpl18^mut^-specific TCRs, we transduced CD8^+^CD3^+^TCR-negative 4G4R thymoma cells with graded concentrations of retrovirus encoding the anti-Rpl18^mut^ TCRs A09, I20, and I02 and plotted TCRβ MFIs versus the percentages of transduced cells (Figure 5, A and B). At equivalent transduction levels, I02 had the highest TCRβ expression (Figure 5B). At similar transduction percentages (Figure 4B), A09 bound dex^Rpl18^ the most avidly (Figure 5, C and D).

To test how these 2 properties combine to influence TCR signaling, we transduced Jurkat reporters with VSV-G–pseudotyped retrovirus encoding A09, I20, or I02 TCRs (Figure 5E). When cocultured with B6WT3 cells pulsed with different Rpl18^mut^ peptide concentrations (Figure 5, F and G), their responsiveness varied over a wide range, with A09 being the most sensitive followed by I20 and I02. We similarly assessed the EC_50_ of additional anti-Rpl18^mut^ TCRs (Figure 5G).

Given that we only cloned 1 anti-ADPGK TCR (A10), we compared it with a published anti-ADPGK TCR that we synthesized and cloned into our expression vector (TCR30; ref. 51) and to the anti-Rpl18 TCR A09. At matched transduction percentages, A10 had higher TCR expression than did TCR30 or A09; nonetheless TCR30 bound dex^ADPGK^ more avidly than did A10 (Figure 5, H and I). Despite weak dex^ADPGK^ binding, a Jurkat line expressing A10 upregulated CD69 when cocultured with B6WT3 cells pulsed with ADPGK^mut^ but not WT ADPGK peptide (Figure 5J).

We similarly analyzed a subset of the anti-Zc3h7b TCRs in 4G4R cells. At matched transduction percentages, TCRs F02, E09, D10, E17, and F02 had comparable TCRβ MFIs, all higher than that of TCR B06 (Figure 5K). When a broader panel of anti-Zc3h7b TCRs was expressed in Jurkat cells and cocultured with Zc3H7b-mutant peptide pulsed B6WT3 cells, we observed a wide range of EC_50_ values (Figure 5L). Reactivity was limited to the mutant peptide in all cases (Supplemental Figure 9).

The anti-PSMD2 TCR G13 was likewise specific for the mutant epitope as it did not react with TMGs 3.9 and 3.23 that were reverted to express WT PSMD2 (Figure 5M). We also obtained the Kerafast MC38 line (MC38K), which exome sequencing revealed to lack the *Psmd2* and *Rpl18* mutations (52). Consistent with this, the anti-Rpl18^mut^ A09 and the anti-PSMD2^mut^ G13 TCR-Jurkat lines only reacted against MC38-Leiden cells (Figure 5N).

### Killing by anti-neoantigen–TCR-transduced T cell products.

**Figure 6:**
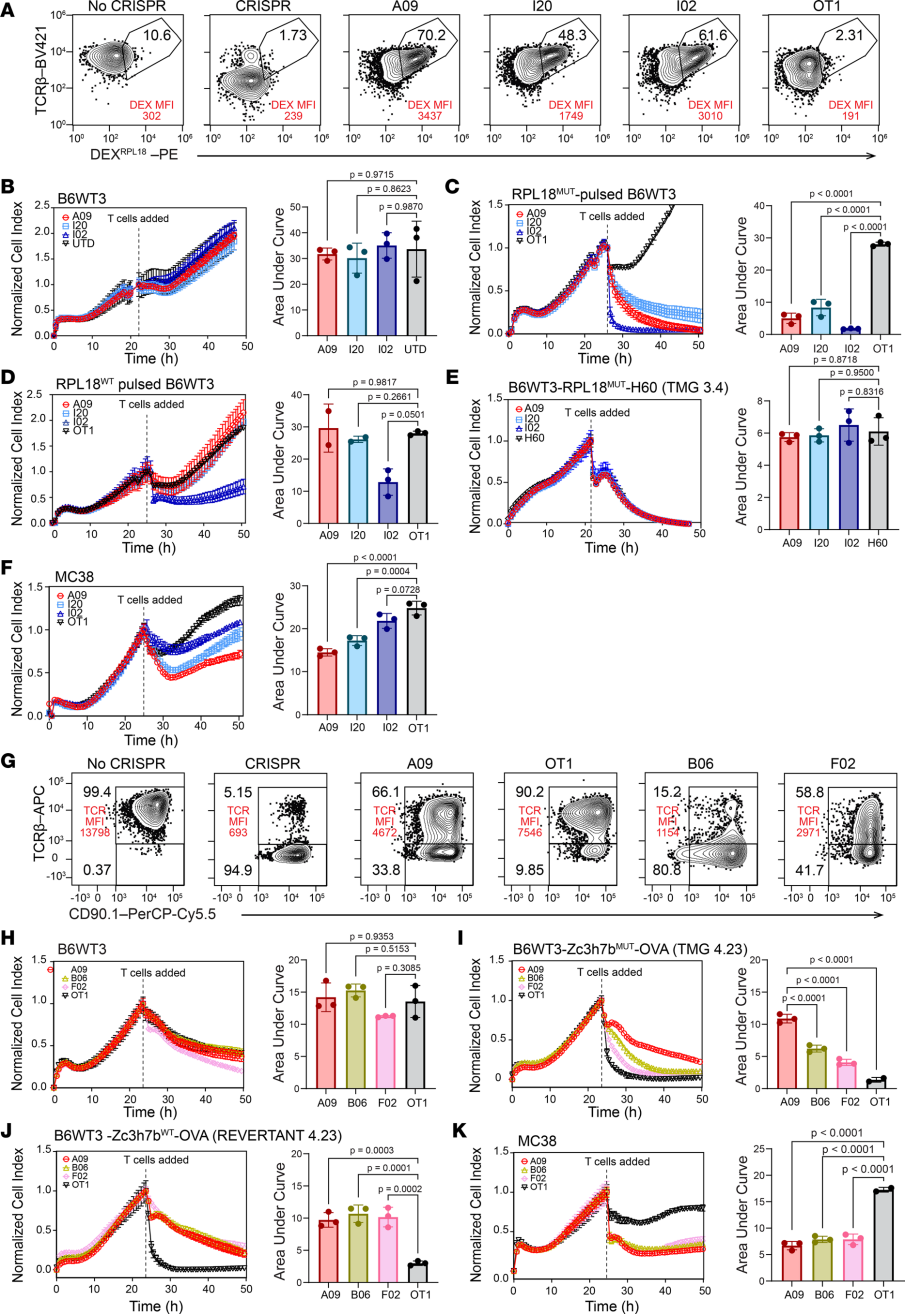
Anti-neoantigen TCR-transduced primary T cells kill neoantigen-expressing targets in vitro. (**A**–**F**) Primary T cell products expressing anti-Rpl18^mut^ TCRs A09, I20, I02, the anti-OVA TCR OT-1, and an anti-H60 TCR were generated using TCX 2.0. These products were assessed by flow cytometry and in vitro cytotoxicity assays. (**A**) Dex^Rpl18^ binding and TCRβ expression, gated on CD8^+^ cells. Killing of (**B**) B6WT3, (**C**) Rpl18^mut^-pulsed B6WT3, (**D**) Rpl18^WT^-pulsed B6WT3, (**E**) B6WT3 cells transduced with a minigene construct expressing Rpl18^mut^ and H60 (**F**) and MC38 cells. (**G**–**K**) Primary T cell products expressing the anti-Zc3h7bmut TCRs B06 and F02, the anti-Rpl18mut TCR A09, and the anti-OVA TCR OT-1 were generated using TCX2.0 and analyzed by flow cytometry (**G**) and in killing assays against (**H**) B6WT3 cells, (**I**) B6WT3 cells transduced with minigenes expressing OVA and Zc3h7bmut, or (**J**) OVA and Zc3h7bWT, or (**K**) unmanipulated MC38 cells. Significance was calculated comparing the AUCs for each condition using 1-way ANOVA followed by Tukey’s post hoc or Dunnett’s multiple-comparison test.

We next made TCX 2.0 TCR products and tested their ability to kill: B6WT3 cells pulsed with peptide targets, B6WT3 cells transduced with cassettes that drive expression of these epitopes, and MC38 cells. Anti-Rpl18^mut^ products expressing TCRs A09, I20, and I02 TCRs did not kill B6WT3 cells or Rpl18^WT^-pulsed B6WT3 cells but did kill Rpl18^mut^-pulsed B6WT3 cells (Figure 6, A–D). Anti-Rpl18^mut^ T cells also killed B6WT3 cells transduced with a cassette expressing the Rpl18^mut^ and H60 epitopes, as did an anti-H60 TCR product (Figure 6E). Importantly, all tested anti-Rpl18^mut^ products killed MC38 cells to a greater degree compared with a control OT-1 TCR product (Figure 6F).

Anti-Zc3h7b^mut^ products (Figure 6G) killed B6WT3 cells transduced with a cassette that expresses Zc3h7b^mut^ and OVA (Figure 6H). This killing was more effective compared with an anti-Rpl18^mut^ product and similar to positive control OT-1–transduced cells (Figure 6I). Killing was specific for the mutant epitope, as there was not killing of targets transduced with a Zc3h7b^WT^/OVA cassette (Figure 6J). Finally, these anti-Zc3h7b^mut^ T cells killed MC38 cells, as did anti-Rpl18^mut^ T cells (Figure 6K). We also tested the functionality of the anti-ADPGK^mut^ A10 and TCR30 TCRs. We made primary T cell products with TCRs A10, TCR30, the anti-Rpl18^mut^ TCR A09, and an anti-SIINFEKL TCR that we cloned (TCR M19). TCR30 cells, and to a lesser extent A10 cells, bound dex^ADPGK^ (Supplemental Figure 10A); only A09-transduced CD8 cells bound dex^Rpl18^. A10, TCR30, and A09 products killed MC38 cells, though A10 killing was relatively weak, consistent with low dex^ADPGK^ binding (Supplemental Figure 10B). M19 and A09 products killed OVA- or OVA-Rpl18 TMG-expressing B6WT3 cells (respectively) whereas A10 and TCR30 cells did not (Supplemental Figure 10, C and D). Reciprocally, A10, TCR30, and M19 T cells killed B6WT3 cells expressing ADPGK^mut^ and OVA, whereas control A09 cells did not (Supplemental Figure 10E). All products had similar effects on untransduced B6WT3 cells (Supplemental Figure 10F). In summary, anti-neoantigen TCRs cloned from TILs redirected primary T cells to kill targets, including MC38 cells, with specificity for the mutant variants.

### Treatment of MC38 tumors with anti-Rpl18^mut^ T cells.

**Figure 7:**
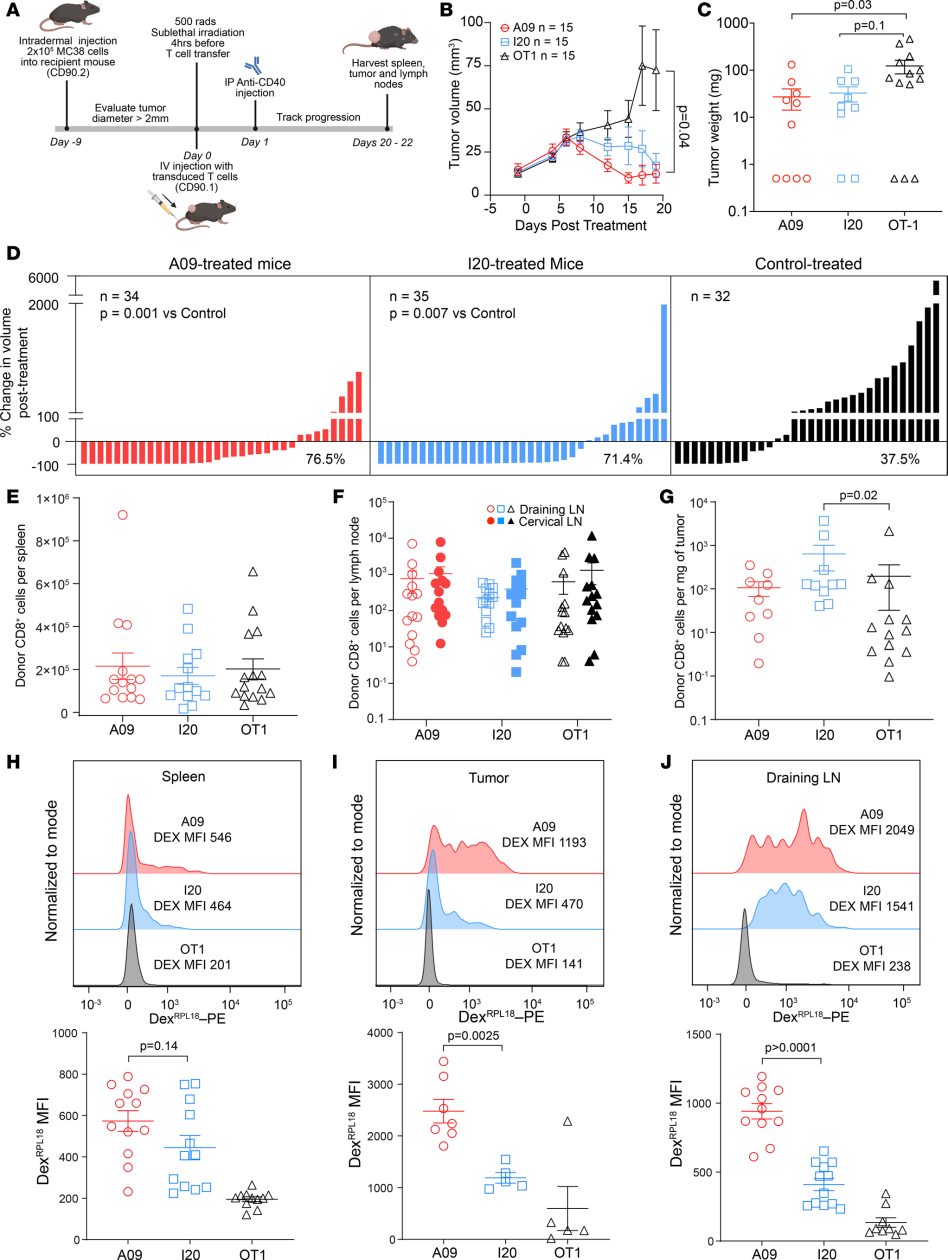
Treatment of MC38 tumors with anti-Rpl18 TCR-transduced T cells. (**A**) Experimental design. MC38 cells were implanted intradermally into CD90.2 B6 mice. Nine days later, mice with tumors more than 2 mm in diameter were sublethally irradiated and injected with CD90.1 TCX 2.0 products transduced with anti-Rpl18^mut^ TCRs A09 or I20 or with the OT-1 TCR, followed by anti-CD40 on day 1. Both A09 and I20 products reduced tumor volumes (**B**) and the weight of the residual tumor (**C**) (significant only for A09; data in **B** and **C** from 1 representative experiment). The lowest values in **C** were too small to be reliably weighed and were assigned arbitrary values. (**D**) Waterfall plots of change in tumor volume from 3 experiments combined. (**E** and **F**) Similar numbers of CD8 cells from the 3 products were recovered from the spleen and draining and contralateral LNs. (**G**) More I20 cells/mg tumor were recovered from the residual tumor bed; only tumors with at least 25 donor CD8 cells were included. (**H**–**J**) Dex^Rpl18^ binding on A09 cells was greater than I02 in tumor and draining LNs (**I** and **J**) but not spleen (**H**). Changes in tumor volume were compared by calculating the AUC for the tumor volume over time and comparing those values using a Kruskal-Wallis test followed by a Dunn’s multiple-comparison post hoc test. Tumor weights were compared by Mann-Whitney rank-sum tests. The number of regressed tumors among groups was compared using a Fisher’s exact test. All other values were compared using Mann-Whitney rank-sum tests.

CD90.2^+^ B6 mice in which the site of MC38 tumor cell injection was greater than 2 mm were sublethally irradiated and infused with B6 CD90.1 TCX 2.0 products expressing anti-Rpl18^mut^ TCRs A09 or I20 or, as a control, the OT-1 TCR. The following day, mice received anti-CD40 as an adjuvant (Figure 7A). Treatment with either I20 or A09 T cells inhibited tumor growth relative to OT-1 cells (Figure 7, B–D, and Supplemental Figure 11A). At euthanization, A09, I20, and OT-1 CD8^+^ T cell numbers were similar in the spleen, draining and contralateral LNs, and tumor bed (Figure 7, E–G, and Supplemental Figure 11). The dex^Rpl18^ MFI on A09 cells was greater than that on I20 cells (Figure 7, H–J), consistent with their properties in reporter cells (Figure 5, C and D) and in the pre-transfer T cell products (Figure 6A). Taken together, these experiments validate our TCR cloning and target-matching processes, in that TCRs cloned from TILs redirected primary T cells into functional therapeutics.

## Discussion

Addressing checkpoint-inhibitor failures in the clinic is a large unmet need. Such failures could be attributable to multiple nonexclusive mechanisms, including an insufficient repertoire or number of T cells specific for neoantigens expressed in tumor cells, immunosuppressive tumor microenvironments, and tumors that have reduced expression of targeted neoantigens or molecules important for T cell recognition and killing (19–26). Some of these causes of treatment failure could potentially be addressed through adoptive immunotherapy with T cell products that target neoantigens. This concept has already been validated to a degree via the use of expanded TILs, which induce responses in patients whose checkpoint inhibitor treatment has failed (30). One can envision that greater efficacy might be achieved with defined T cell products genetically manipulated to target specific neoantigens (3, 31, 53). Such products would also be amenable to engineering to improve efficacy in an immunosuppressive tumor microenvironment. Creating this type of therapy requires technologies to enable the rapid cloning of TCRs from tumor-infiltrating CD8 cells, the identification of their neoantigen specificities, and the selection of quality receptors based on in vitro properties. This process would need to be sufficiently rapid to match the pace of disease progression and be achievable with the size of tumor biopsies likely to be acquired. Finally, widespread implementation would require the costs to be sufficiently low to be sustainable with broad application.

In the present manuscript, we describe the development of such a process in a mouse tumor model. From MC38 tumors, we screened 295 TCRs from infiltrating CD8 cells against 212 potential neoantigens, which led to the identification of multiple TCRs reactive against peptides derived from tumor-specific mutations in Rpl18 or Zc3h7b, along with single TCRs against peptides from PSMD2 or ADPGK. To accomplish this, we combined multiple enabling technologies: (a) the amplification of TCRα and TCRβ variable regions from single cells in a way that permits single-step cloning into a retroviral backbone incorporating TCRα and TCRβ constant regions, all without expensive and time-consuming nucleic acid sequencing or gene synthesis; (b) application of robotic pipetting; (c) development of cell lines for the sensitive screening of TCR reactivities; (d) an artificial APC and methods to stably transduce it with TMG; (e) a multiplexed method for screening reporter cells against APCs; and (f) methodologies for adoptive primary T cell product generation that incorporate endogenous TCRα and TCRβ knockdown. Importantly, we took this process through to the efficacious treatment of MC38-bearing mice with cloned anti-neoantigen TCRs. Taken together, this body of work demonstrates a therapeutic workflow and suggests that such an approach could be feasible in the clinic.

A strategy for cloning TCRs from single cells using nested PCR and Gibson assembly was previously reported (54, 55). However, for several reasons, TCXpress is simpler and capable of a higher throughput. Critically, the previously reported approach requires next generation sequencing of the TCRα amplicons. This is because the first-round amplification does not capture the entire 5′ TRAV coding region. A further consequence of this strategy, in addition to the need for sequencing, is that each specific amplified 3′ portion of the TRAV must be inserted into 1 of 13 vectors encoding the TRAV family–specific 5′ portion that was not cloned. In contrast, our approach does not require sequencing, as it amplifies the entire TCRα and TCRβ variable regions, which enables direct Gibson cloning of the second-round amplicons into a single accepting vector.

scRNA-Seq can also enable the generation of TCRα and TCRβ chains from single cells. However, a TCR-screening pipeline based on such sequencing data would require obtaining high-quality TCR sequences from each single cell, synthesis of gene blocks for each TCRα/TCRβ pair, followed by cloning into the expression vector. The cost of sequencing and gene-block synthesis is far greater than with the TCR cloning pipeline we describe. It is true that our approach requires a second deconvolution step; however, this only needs to be performed on TCRs that are positive in screening assays, whereas the scRNA-Seq approach requires gene-block synthesis for all TCRs. Another approach could be to start with scRNA-Seq data and only pursue the most expanded clones. However, there is no guarantee that neoantigens drive such highly expanded clones nor that highly expanded clones are only driven by neoantigens; in fact, this is unlikely to be the case. Hence, the strategy of gene-synthesizing only expanded clones would risk not capturing high-value TCRs that might be less frequent, a risk that is magnified by the limited sampling. Moreover, strategies to target neoantigens may require therapeutics against multiple neoantigens to capture the full tumor clonal architecture (11, 56), and therefore a broader unbiased approach may be superior.

Regardless of the approach for cloning TCRs, an important challenge is developing ex vivo pipelines for determining which TCRs against a given neoantigen target will be the most efficacious in vivo (57), which may not easily be inferred from standard avidity measurements (58, 59).

The process of unbiased cloning of TCRs from TILs and screening them against putative neoantigens also provided insights into the nature of spontaneous antitumor T cell immunity. The predominant target specificity we identified in MC38 tumors was for Rpl18^mut^, which was not unexpected given prior published tetramer staining data (37). Nonetheless, Rpl18 was ranked as a potential neoantigen independent of prior publications, and our approach cloned TCRs against it without the use MHCI-multimers or peptide vaccination (37). We characterized 10 anti-Rpl18 TCRs isolated from 3 mice and as many as 5 unique TCRs from a single mouse, and these TCRs had a range of functional avidities. The apparent immunodominance of Rpl18 could in part be driven by a high level of mRNA expression (37, 41). This epitope may also have a high precursor frequency of reactive T cells, as inferred by the broad range of CDR3 sequences and Vβ and Vα family members used in the 10 TCRs we cloned. In a second campaign, we cloned 9 TCRs against Zc3h7b from 3 different tumors with 5 TCRs from a single tumor, again with a range of functional avidities. Notably, all used TRAV12-2 but they did not have a TRBV bias. Zc3h7b is also highly expressed in MC38 cells (41). In contrast to Rpl18 and Zc3h7b, we only isolated single TCRs against PSMD2 and ADPGK. Both *Psmd2* and *Adpgk* are well expressed at the RNA level (37, 41) and the epitopes against which we found TCRs are predicted to be presented. Both Yadav et al. and Hos et al. predicted ADPGK as being immunogenic neoantigens in MC38 cells (37, 41). Yet, vaccination against ADPGK yielded only a small response relative to that against Rpl18^mut^ (only identified by Hos; ref. 37) and a prior campaign to clone TCRs against ADPGK only yielded a single TCR (51). This comparative analysis highlights how difficult it is to predict immunogenicity based on RNA expression and estimates of MHCI binding. Lacking better methods to predict immunogenicity, approaches such as ours that enable the parallel screening of many TCRs against many potential neoantigens should be valuable for studies of T cell responses and the clinical translation of T cell immunotherapies targeting neoantigens.

Surprisingly, from our first campaign, we identified and characterized 7 unique TCRs that were reactive against B6 background B6WT3 cells and MC38 cells, with 1 of these TCRs identified 6 times, reflecting intratumoral expansion of T cells with this TCR. We did not attempt to identify individual autoreactive TCRs from all TCR pools that demonstrated autoreactivity (including those that were autoreactive in our second campaign) and hence, the 7 TCRs found among the 295 screened is most likely an underestimate. Nonetheless, these data suggest that antitumor immunity naturally includes substantial autoreactivity in addition to neoantigen reactivity. CD8 cells recognizing nonmutant proteins including the tumor testes antigens (i.e., MAGE and NY-ESO1) have long been described, but the expression of these cells is relatively tumor restricted (60, 61). Similarly, responses to nonmutant versions of melanoma and melanocyte-restricted targets are also common (62). Again, these cells have a relatively restricted tissue expression. There have been fewer descriptions of CD8 cells reacting against more ubiquitously expressed antigens, as is likely the case with at least some of the autoreactive TCRs we isolated (8, 63). The degree to which these autoreactive TCRs contribute to tumor control, their ontogeny in the tumor, and whether they pose a risk of autoreactivity are all interesting areas for future investigation. Patients with certain types of cancers can develop autoimmune syndromes (64–67), and checkpoint blockade induces autoimmunity in a substantial fraction of patients. Perhaps autoreactive T cells elicited as part of the antitumor response contribute to one or both of these scenarios. A future direction would be to transduce primary T cells with autoreactive TCRs, such as the ones we isolated, and determine whether they can direct killing and in vivo toxicity.

The failure to identify targets for 260 of the 295 screened TCRs despite screening against more than 200 predicted neoantigens is of interest. It could reflect that many intratumoral T cells are not tumor-reactive and represent a “sympathetic infiltrate” that migrated into an inflammatory site, which was in turn driven by the T cells that are tumor-specific or autoreactive, as has been shown in autoimmunity (68). Nonetheless, there could have been multiple nonexclusive technical factors that impeded identification of targets for TCRs that were truly specific for the tumor. First, our screening approach may be insufficiently sensitive. As illustrated by the molecular deconvolution data from positive multiplexed cultures, the amplification and cloning processes can create damaged or incomplete TCR chains that generate suboptimal TCRs. Further, some T cells express a second TCRα chain, which, when paired to the TCRβ, will not be reactive. This dilution could result in some mixed TCR screening cultures having too few Jurkat cells with the correct TCR to lead to sufficient CD69 expression for the culture to be scored as positive. This could be especially problematic for relatively weak TCRs or for epitopes that bind less stably to MHCI on APCs or tumor cells.

Another potential source of inefficiency is that some target neoantigen epitopes embedded in the minigene might not be effectively processed. Although others have reported that MHCI-restricted antigens are well-presented in tandem minigene constructs (69), in screening a wide variety of previously uncharacterized epitopes, it would be challenging to validate that each is expressed. In preliminary work, we did test various linker sequences between the minigenes as well as determine that there were not obvious positional effects within the TMG. We further tried to mitigate this issue by screening each minigene twice in 2 different positions. Overall, although this could be a reason why some epitopes were not effectively screened, it is unlikely to explain much of the failure to identify targets for a large number of TCRs.

Our target libraries may also have been incomplete. Current neoantigen calling focuses on nonsynonymous coding or frameshift mutations that result in a potentially longer non-self tract of aa. Algorithmic predictions about antigen processing and peptide generation and the binding affinities of the resultant peptides to specific MHCI molecules are then used to filter peptides for further evaluation. This approach may leave out peptides that are not predicted to be either processed or bind MHCI, but in fact actually do so, thus precluding identification of T cells that recognize them. Focusing on TCRs from expanded clones from exhausted T cells in TILs may enrich for tumor-reactive TCRs and improve the efficiency of matching such TCRs to neoantigen targets (8). An improved understanding of the rules for immunogenicity will require the deciphering of more TCR/MHC-peptide matches, and the TCXpress process, if broadly applied, could contribute to this dataset. Recently, there have been advances in using artificial intelligence to approach this task (70).

Conventional neoantigens may not be the only TCR targets. Polypeptides generated from noncanonical mRNAs uniquely expressed in cancer cells could generate immunogenic epitopes (71–74). In the case of the MC38, T cells responding to endogenous retroviral (ERV) gene p15e were found to be expanded in TILs (75, 76) and have been suggested as potential target antigens more broadly (77–79). Other sources of noncanonical peptides include novel transcripts that are generated via tumor-specific aberrant gene regulatory networks, transcripts from noncoding regions of DNA, and altered splice variants (71, 80). In the future, application of the TCX platform to the generation of TCRs that recognize normally suppressed ERV products, or other “dark matter” peptides, could lead to a T cell therapeutic applicable to multiple cancer types.

TCXpress has also been applied to clone TCRs from human T cells and generated a TCR targeting the human minor histocompatibility antigen HA-1 (81); that TCR is currently in a clinical trial (ClinicalTrials.gov NCT06704152). It also has been used to clone multiple receptors against the minor histocompatibility antigen HA-2 in a patient with graft-versus-host disease (81). Finally, we believe that the TCXpress process could be used to isolate anti-neoantigen TCRs from human specimens, which could ultimately lead to novel therapeutics.

## Methods

### Sex as a biological variable

Since MC38 cells are female, we used female recipient mice and donor mice for creating T cell products. Because the B16 model system was developed to create products for treating MC38 tumors, we also used female donor and recipient mice.

### Cell lines

See Supplemental Methods.

### Antibodies

See Supplemental Table 3.

### Mice and tumor models

#### Mice.

C57BL6 (CD90.1 Strain # 000406 and CD90.2 Stock # 000664) mice were purchased from The Jackson Laboratory.

#### Tumor establishment and treatment.

B16, B16-MG, or MC38 cells were injected intradermally. Tumors were measured at least twice every 5 days by 2 investigators blinded to treatment group assignments. Adoptive T cell products were injected i.v. Mice with tumors more than 2 mm in diameter were assigned to treatment groups such that the average tumor diameters for each group were similar. Prior to T cell infusions, mice received 500 cGy of total body irradiation (cesium source). Eighteen hours after T cell infusion, mice received 50 μg i.p. of the anti-CD40 antibody FGK45 (BioXcell).

### Isolation and analysis of TILs

Dissected tumors were minced in DMEM (Gibco) followed by centrifugation. Pellets were digested with 400 U/mL collagenase D and 40 U/mL DNase I (Sigma-Aldrich) at 37°C for 30 minutes. Digested tumors were filtered and washed before being resuspended in PBS for antibody staining. Intracellular staining was carried out using an Invitrogen Transcription Factor kit.

### Reverse transcription, TCRα/β amplification, and TCR reconstruction

CD8^+^ TIL cells were single cell–sorted into 384-well PCR plates. The cell in each well was lysed, followed by cDNA synthesis using random hexamers (Promega) and Superscript IV (Invitrogen). After cDNA synthesis, first-round PCR amplifications of TCRα/β variable regions were performed using a custom primer cocktail (Supplemental Table 1). Products from each well were aliquoted to two 384 well plates using a BioMek i7 liquid handle (Beckman Coulter), and TCRα and TCRβ variable regions were separately amplified using nested primers with anchor sequences for Gibson assembly (82) (Supplemental Table 1), purified with Sera-Mag Speed Beads (Cytiva), and quantified by SYBR green (Supplemental Figure 1). Using a BioMek i7, the TCRα and TCRβ products were mixed with a modified TCRα constant region (Supplemental Table 4) and a murine stem cell virus-based retroviral vector containing a modified TCRβ constant region (Supplemental Table 4) followed by Gibson assembly using Gibson assembly master mix (New England Biolabs). *E*. *coli* were transformed with each Gibson product and grown in carbenicillin-containing broth. Plasmids were purified from the broth cultures using Cytiva DNA-binding membranes.

See Supplemental Methods for the creation of TCR-expressing retrovirus and virus titering, Jurkat cell transduction, prediction of MC38 neoantigens, and pairing of TCRs with cognate antigen.

TMGs encoding up to 8 neoantigens flanked by K^b^-presented epitopes from H60 (LTFNYRNL; carboxy terminus) and OVA (SIINFEKL; amino terminus) (38, 39) were synthesized (Integrated DNA Technologies). Individual neoantigen minigenes encoded 25 aa and centered on the changed residue. For insertions, deletions, and frameshift mutations, minigenes included up to 12 aa upstream and at least 12 aa downstream of the mutation. Gene blocks were cloned into retroviral backbones (Supplemental Figure 2). Single construct retroviral supernatants were generated, and B6WT3 cells were transduced. Puromycin-selected B6WT3-TMG cell lines were treated with IFN-γ (10 μg/mL) for 48 hours and seeded into 96-well flat-bottom plates (2.5 × 10^4^ cells/well) in cRPMI. The IFN-γ–containing media was removed, and TCR-transduced Jurkat cells were added to each to well. Cells were incubated for 18 hours; stained with antibodies against mouse TCRβ, human CD8, and human CD69; and analyzed by flow cytometry (Figure 2 and Supplemental Figure 3).

See Supplemental Methods for molecular deconvolution of TCR-encoding plasmids and transduction of primary T cells and killing assays. TCR sequences of deconvoluted TCRs are in Supplemental Table 5.

### Statistics

Statistics for specific experiments are included in figure legends. Differences between groups were calculated by 1-way ANOVA with either a Tukey’s post hoc or Dunnett’s multiple-comparison test or with a Mann-Whitney rank-sum test. Significance of tumor regression was calculated using a Fisher’s exact test. A *P* value of less than 0.05 was considered significant.

### Study approval

All animal experiments were approved by the University of Pittsburgh IACUC.

### Data availability

MC38 DNA sequencing data have been deposited in NCBI’s Gene Expression Omnibus (GEO GSM9625251). All other data are available upon request to the corresponding author. Values for all data points in graphs are reported in the Supporting Data Values file.

## Author contributions

MJS and WDS conceived of the project, obtained funding, designed experiments, supervised the work, analyzed data, and wrote the manuscript. AMR led the design and execution of experiments, performed experiments, analyzed data, supervised other scientists, analyzed data, and wrote the manuscript. SC, WW, LGD, KT, JGS, CS, CP, and KC performed experiments and analyzed data. LGD also created manuscript figures. AM and KT analyzed sequencing data. DW provided technical advice and created the Jurkat reporter cells.

## Conflict of interest

MJS, WDS, and AMR have equity interests in BlueSphere Bio. MJS and WDS were compensated consultants for BlueSphere Bio.

## Funding support

- University of Pittsburgh Medical Center Immune Therapy and Transplant Center.
- BlueSphere Bio.

## Supplementary Material

Supplemental data

Supplemental table 1

Supplemental table 2

Supplemental table 3

Supplemental table 4

Supplemental table 5

Supporting data values

## Figures and Tables

**Table 1:**
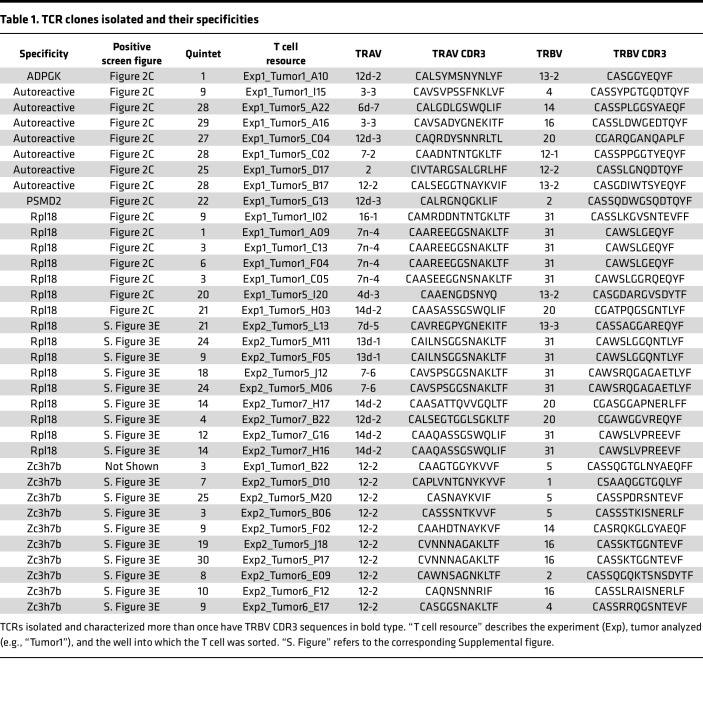
TCR clones isolated and their specificities

## Version 1 04/28/2026

In-Press Preview

## Version 2 06/22/2026

Electronic publication
