# Supplementary material for: A platform for parallel TCR cloning and testing enables anti-neoantigen tumor immunotherapy
Source: JCI Insight. 2026 Apr 28;11(12):e203622. doi: 10.1172/jci.insight.203622 (PMC13313532; doi:10.1172/jci.insight.203622)

**Supplemental Methods**

**Cell lines**

B16-F10 tumor cells lines were obtained from the ATCC (Catalog # CRL-6475**)**. These were further modified to express a tandem minigene encoding epitopes from gp100 (EGSRNQDWL), H60 (LTFNYRNL), TRP1 (TAPDNLGYM), TRP2 (SVYDFFVWL) and OVA (SIINFEKL) as described in in Supplemental Figure 6 (B16-MG cells). Platinum-E (PLAT-E; Catalog # RV-101) and Platinum-GP (PLAT-GP; #RV-103) retrovirus packaging lines were purchased from Cell Biolabs (San Diego, CA). MC38 tumor cells(1) were a gift from Lary Kane (University of Pittsburgh). We modified 4G4 thymoma cells (2) to create cells that report TCR signaling (4G4R). TCR$\alpha$/$\beta$-knockout human CD8$\alpha^+$ Jurkat reporter cells were obtained from BlueSphere Bio. B6WT3 mouse embryonic fibroblast cells(3) were obtained from Robert Hendrick (University of Pittsburgh).

**Culture media**

Complete RPMI (cRPMI; used for all cultures that included T cells and MC38 cells) is RPMI (Gibco) supplemented with 10% FBS (Hyclone), NaPyruvate 1mM, non-essential amino acids, L-glutamine 2mM, Hepes 10mM and 2-beta-mercaptoethanol 50mM. PLAT-E media (used for all retrovirus generation) is DMEM with L-glutamine (Gibco), 10% FBS, NaPyruvate 1mM and 1x nonessential amino acids.

**4G4 cell modifications**

Briefly, 4G4 cells which do not express TCR$\alpha$ or TCR$\beta$ chains were transduced with two retroviruses, one encoding murine CD3$\alpha$, CD3$\varepsilon$, CD3$\gamma$ and CD3$\zeta$ chains linked to ametrine (poly-CD3; gift of Dario Vignali (4)) and the second expressing mouse CD8$\alpha$. Ametrine$^+$ CD8$\alpha^+$

cells were purified by FACS, and subcultures were selected based on rapid growth and high levels of surface CD8 expression.

**Calculations of tumor volumes**

Tumor volume was determined using the formula: ½*(Length *Width$^2$)

$$\text{Volume Elipsoid: } \frac{Length\ x\ Width^2}{2}$$

(Where L is length and W is width of the tumor). Percent change in tumor volume was calculated with the following formula:

% Change in tumor volume= (Tumor volume at the end of treatment-tumor volume on the start of treatment/ tumor volume on the start of treatment) *100

$$\%\ \text{Change in Tumor Volume} = \frac{Tumor\ Volume\ at\ "day\ 20" - Tumor\ volume\ at\ "day - 1"}{Tumor\ volume\ at\ "day\ \ - 1"}\ x\ 100$$

Mice were euthanized if the tumor diameter exceeded 20mm, if they had lost >20% of their starting body weight on 2 consecutive measurements, or if they were moribund.

**Creating TCR-expressing retrovirus.**

*VSV-G pseudotyped virus for Jurkat transduction.* MSCV-based plasmids containing TCRα and TCRβ chains (1 μg/well) were individually co-transfected into PLAT-GP cells (1.5x10$^5$ cells/well) with VSV-G-expressing vector (1 μg/well) using 2 μL/well LipoJet™ (SignaGen #SL100468) in 24-well plates. 48 hours later, supernatants were collected.

*Ecotropic retrovirus production for primary T cell transduction*. 5x10$^6$ PLAT-E cells were seeded in T75 flasks. At 80% confluency, the media was replaced with 10mls complete RPMI (cRPMI) and the cells were transfected with 20μg TCR-expressing plasmid using the LipoJet™ In Vitro Transfection Kit (Ver. II). 18 hours later, the media was replaced with 10mL cRPMI and the cells

were incubated for an additional 30 hours. At 48 hours post-transfection, media was harvested, and debris was cleared through centrifugation. RetroX viral concentration reagent (Takara) was added to the supernatant and virus was concentrated according to the manufacturer's instructions.

**Virus titering**

4G4R cells were infected with dilutions of virus stock in 3μg/ml polybrene and centrifuged at 1000g for 90 minutes at 37° C. Cells were returned to the incubator and media was changed 18 18 hours later. Titers were determined based on the expression of mCherry or TCRβ 48-72 hours later (see Supplemental Figure 7).

**Jurkat cell transduction**

VSV-G-pseudotyped retroviral supernatants were added to Jurkat cells ($1x10^5$ cells/well) with 4 μg/mL polybrene (Sigma-Aldrich #107689) and centrifuged at 37°C for 90 min without braking after which the cells were returned to the incubator. Media was changed in next day. To puromycin-select transduced cells, puromycin was added to the media (2μg/ml) for 48 hours and then changed with fresh cRPMI media for cell expansion.

**Neoantigen prediction**

Whole genome sequencing (WGS) and sequence alignments of MC38 cells were carried out by the University of Pittsburgh Genomics Core. This initial alignment was then refined by GATK base recalibration of insertions and deletions (5), followed by indel realignment and duplicate removal, all following GATK best practices recommendations (5,6). Variant calling was performed on WGS data using three variant callers: Mutect, Mutect2, and Strelka (7,8). This multi-tool approach aimed to minimize false positives inherent in variant-calling algorithms.

Variants identified by at least two out of the three tools were annotated using Ensembl Variant Effect Predictor(9). Variants resulting in an amino acid change were selected, yielding 807 neoantigen candidates. NetMHCPan 4.0 was employed to estimate the EC50s of these predicted peptides to H-2K$^b$ or H-2D$^b$ (10). Using a publicly available bulk RNA-Seq dataset for MC38 cells (11), the transcripts per million (TPM) for each gene encoding potential neoantigens were calculated using STAR aligner (12) and geneCount (Illumina), so as to be able to incorporate gene expression into choosing putative neoantigens to pursue. TPM, predicted epitope binding scores, and variant allele fractions were each converted to percentile ranks across the candidate neoantigen dataset to place all features on a comparable scale. These normalized values were then integrated using a weighted linear scoring framework to generate a composite score reflecting the predicted likelihood that a candidate neoantigen will elicit a CD8$^+$ T cell response.

**Molecular deconvolution of TCR-encoding plasmids.**

After a TCR expressed in Jurkat cell line was found to be desirable, the sequences and specificity of the TCR were confirmed by the "molecular deconvolution" of the vectors. Briefly, plasmid generated from the bulk E. coli cultures from the original Gibson assembly product was transformed into bacteria, followed by plating on agarose plates. Six to eight colonies were selected, expanded, and plasmid DNA was isolated. The plasmids were sequenced and used to create individual lentivirus supernatants. Jurkat reporter cells were transformed, reselected, and screened against B6WT3 expressing the appropriate antigen.

**Sanger sequencing of TCRβ PCR products and TCR retroviral vector plasmids**

Bead-purified second round TCRβ chain amplification products were Sanger sequenced directly with a single primer (GAAGAAAACCCCGGTCCC) without subcloning (Azenta). The TCRα and

TCRβ regions of single colony-derived plasmids were sequenced with the same primer used to sequence purified TCRβ chains plus three other primers (TTGGCTTTTGACCCCC, AGGTTCTGGGTTCTGGATGT, GGAGTCACATTTCTCAGATCCT) so as to cover all Gibson insertions.

**Transduction of primary T cells**

T cells were purified from C57BL6 mice splenocytes using Easy Sep Mouse T cell negative selection kits (Stem Cell, cat No. 19851). For TCX 1.0, on day 0, purified T cells were cultured at a 1:1 ratio with anti-CD3/CD28 Dynabeads (Thermo-Fisher/Gibco, cat. No. 11453D) in cRPMI with 50U/ml human IL-2 (Peprotech) for 48 hours at 37°C. For TCX2.0, T cells were activated in a 12 well plate with 3μg/well plate-bound anti-CD3 (clone 2C11, Biolegend, cat no. 100302) with soluble anti-CD28 (2μg/ml, clone 37.51, Biolegend, cat No. 102116), and anti-CD137 (10μg/ml, clone 3H3, InvivoMAb Bio-X Cell, cat. No BE0239) in cRPMI with 50U/ml IL-2 for 48 hours at 37°C. After 48 hours, activated T cells were harvested and subjected to endogenous TCR α/β knockout using the Stem Technologies CRISPR-Cas9 ARCITect system (Stem Cell Technologies, Vancouver, British Columbia, CA). In brief, crRNA containing sequences targeting the TCRα constant region (GAGACCGAGGATCTTTTAAC) and the TCRβ constant region (GCCCCTGGCCAAGCACACGA) and Tracr RNA complexes were prepared as per the manufacturer's protocol. gRNA-Cas9 complexes were prepared by combining Cas-9 (Stem Cell, cat no. 76004) with crRNA-Tracr RNA complexes followed by electroporation of activated T cells using with Lonza electroporation system and the P3 cell solution kit (Cat.no PB P3-U2250). Following electroporation, cRPMI with IL-2 (50U/ml) was added to the cells which were rested for 1hr at 37°C. These electroporated T cells were then transduced with matched titers (10 infectious units per cell) of retroviral supernatants encoding CRISPR-resistant TCRs of interest.

Transduced cells were washed the next day and expanded in cRPMI and IL-2 (50U/ml) for 4 additional days. Endogenous TCR knockout and TCR transduction was measured by flow cytometry by staining cells with antibodies against CD8, TCRβ and using MHC-multimers when appropriate. Functional assays and cell transfer for ACT were performed at day 8 post-activation.

**T cell killing assays**

Target specific killing by engineered T cells was analyzed using the xCELLigence system (Agilent). Published protocols were adapted for use with B6WT3, MC38 and B16-derived cell lines. Prior to being plated all target cell lines were pretreated with IFN-γ. In brief, xCELLigence gold E-plates were blocked for 1 hour at 37°C with 0.1% BSA in PBS. Plates were washed twice with PBS, media was added and allowed to equilibrate at 37°C, followed by the addition of cells (in triplicate for each condition). Adhesion and growth were recorded on the xCELLigence reader for 24 hrs. For MC38 killing assays, 80,000 MC38 cells were added the day prior to adding $10^5$ live cells for each TCR-modified product. For killing of B6WT3 cells, 25,000 B6WT3 cells were seeded the day prior to adding $10^5$ live cells for each TCR-modified product. For peptide-specific killing assays, the cell layers were pulsed with peptide and returned to the xCELLigence for 1-2 hours prior to the addition of T cells and incubation for 24 hours at 37°C. Relative cell indices were calculated based on dividing the conductance at each time-point by the maximum conduction during the experiment. Areas under curve were calculated for each type of treatment. Statistical analysis was done by one-way ANOVA.

**References**

1. Corbett TH, Griswold DP, Jr., Roberts BJ, Peckham JC, Schabel FM, Jr. Tumor induction relationships in development of transplantable cancers of the colon in mice for chemotherapy assays, with a note on carcinogen structure. Cancer Res **1975**;35(9):2434-9.

2. Hong SC, Chelouche A, Lin RH, Shaywitz D, Braunstein NS, Glimcher L*, et al.* An MHC interaction site maps to the amino-terminal half of the T cell receptor alpha chain variable domain. Cell **1992**;69(6):999-1009 doi 10.1016/0092-8674(92)90618-m.
3. Flyer DC, Pretell J, Campbell AE, Liao WS, Tevethia MJ, Taylor JM*, et al.* Biology of simian virus 40 (SV40) transplantation antigen (TrAg). X. Tumorigenic potential of mouse cells transformed by SV40 in high responder C57BL/6 mice and correlation with the persistence of SV40 TrAg, early proteins and sequences. Virology **1983**;131(1):207-20 doi 10.1016/0042-6822(83)90546-9.
4. Holst J, Wang H, Eder KD, Workman CJ, Boyd KL, Baquet Z*, et al.* Scalable signaling mediated by T cell antigen receptor-CD3 ITAMs ensures effective negative selection and prevents autoimmunity. Nat Immunol **2008**;9(6):658-66 doi 10.1038/ni.1611.
5. McKenna A, Hanna M, Banks E, Sivachenko A, Cibulskis K, Kernytsky A*, et al.* The Genome Analysis Toolkit: a MapReduce framework for analyzing next-generation DNA sequencing data. Genome research **2010**;20(9):1297-303 doi 10.1101/gr.107524.110.
6. DePristo MA, Banks E, Poplin R, Garimella KV, Maguire JR, Hartl C*, et al.* A framework for variation discovery and genotyping using next-generation DNA sequencing data. Nat Genet **2011**;43(5):491-8 doi 10.1038/ng.806.
7. Cibulskis K, Lawrence MS, Carter SL, Sivachenko A, Jaffe D, Sougnez C*, et al.* Sensitive detection of somatic point mutations in impure and heterogeneous cancer samples. Nat Biotechnol **2013**;31(3):213-9 doi 10.1038/nbt.2514.
8. Saunders CT, Wong WS, Swamy S, Becq J, Murray LJ, Cheetham RK. Strelka: accurate somatic small-variant calling from sequenced tumor-normal sample pairs. Bioinformatics **2012**;28(14):1811-7 doi 10.1093/bioinformatics/bts271.
9. McLaren W, Gil L, Hunt SE, Riat HS, Ritchie GR, Thormann A*, et al.* The Ensembl Variant Effect Predictor. Genome Biol **2016**;17(1):122 doi 10.1186/s13059-016-0974-4.
10. Jurtz V, Paul S, Andreatta M, Marcatili P, Peters B, Nielsen M. NetMHCpan-4.0: Improved Peptide-MHC Class I Interaction Predictions Integrating Eluted Ligand and Peptide Binding Affinity Data. J Immunol **2017**;199(9):3360-8 doi 10.4049/jimmunol.1700893.
11. Hos BJ, Camps MGM, van den Bulk J, Tondini E, van den Ende TC, Ruano D*, et al.* Identification of a neo-epitope dominating endogenous CD8 T cell responses to MC-38 colorectal cancer. Oncoimmunology **2019**;9(1):1673125 doi 10.1080/2162402X.2019.1673125.
12. Dobin A, Davis CA, Schlesinger F, Drenkow J, Zaleski C, Jha S*, et al.* STAR: ultrafast universal RNA-seq aligner. Bioinformatics **2013**;29(1):15-21 doi 10.1093/bioinformatics/bts635.

**Supplemental Figure 1. Representative amplified TCR$\alpha$ and TCR$\beta$ variable region products.** (A) TCR$\alpha$ products after the second-round amplification, pre- and post-purification. (B) Using a SYBR green assay and a 476 base pair standard, cures were generated determine TCR DNA concentrations. (C, D) Representative purified TCR$\alpha$ and TCR$\beta$ concentrations determined by SYBR green emission from a TCXpress purification run. Each square on each TCR$\alpha$ (C) and TCR$\beta$ (D) plate represents purifications from products derived from a single cell.

**Supplemental Figure 1. Representative amplified TCRα and TCRβ variable region products.**

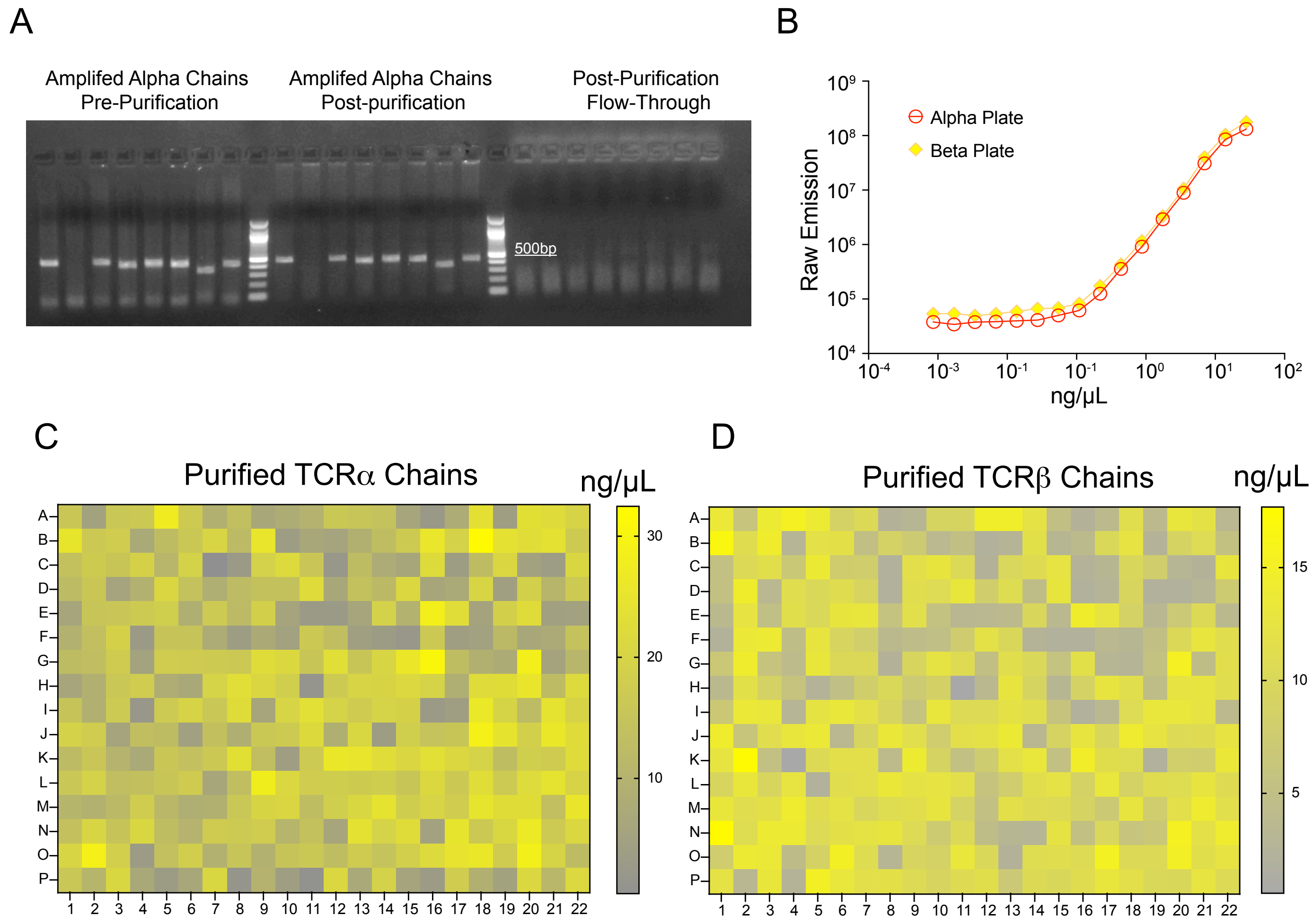

**Supplemental Figure 2. Creation of TMG-transduced B6WT3 APC lines.** B6WT3 cells were transduced with TMG-encoding retrovirus (A) wherein the TMG construct also encodes SIINFEKL. $K^b$:SIINFEKL surface presentation was detectable on transduced B6WT3 cells pre- and post-puromycin selection by the 25D1 antibody (B). Putative neoantigens were assembled into two assemblies (Assemblies 1 and 2), with each predicted neoantigen in a different context in each (C). IFN-$\gamma$ treatment of parental B6WT3 cells, TMG-modified B6WT3 cells and MC38 cells led to upregulation of $K^b$ and $D^b$ (D).

**Supplemental Figure 2. Creation of TMG-transduced B6WT3 APC lines.**

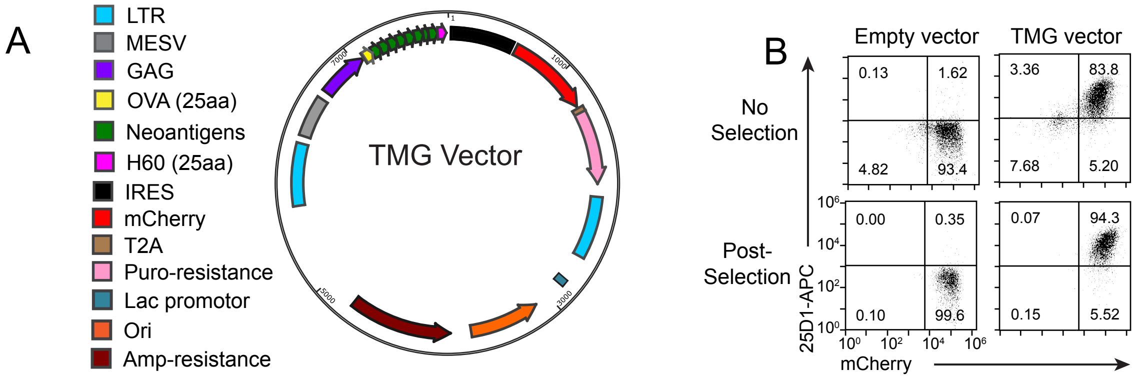


C

Assembly 1

| Minigene | Position 2 | Position 3 | Position 4 | Position 5 | Position 6 | Position 7 | Position 8 | Position 9 |
|---|---|---|---|---|---|---|---|---|
| 3.0 | Reps1 | Aatf | Rpl18 | Irgq | Dpagt1 | Wbp11 | Adpgk | Cpne1 |
| 3.1 | Slc35c1 | Tpbg | Sars | Kif14 | Large1 | Phldb2 | Hacd2 | Nrbp2 |
| 3.2 | Ghr | Hacd1 | Fads1 | N4bp2l2 | Srebf1 | Ppp1r7 | Ganab$_{733}$ | Gtf2i |
| 3.3 | Psmd2$_{822}$ | Rad50 | Slc25a45 | Tango6 | Eif4e2 | Eng | Ndc1 | Irgq |
| 3.4 | Rpl18 | Aldh3a2 | Srebf2 | Cand1 | Gba2 | Fcsk | Ndufs6 | Abcb8 |
| 3.5 | Kmt2b | Arhgap12 | Med1 | Tdg | Ankrd13a | Map1a | Adat1 | Zfp984 |
| 3.6 | Cplane1 | Prxl2a | Pop1 | Ndrg1 | Med12 | Adpgk | Sorbs1 | Paip1 |
| 3.7 | Ogfr | Senp5 | Cpne1 | Kcnv1 | Obscn | Skp2 | Pdia5 | Trio |
| 3.8 | Pi4kb | Dpagt1 | Ganab$_{394}$ | Raly | Agtpbp1 | Snap47 | Synj1 | Specc1 |
| 3.9 | Btg1 | Tet3 | Lmo7 | Psmd2755 | Retreg1288 | Cryzl1 | Myo15 | Get3 |
| 3.10 | Zbtb40 | Map7d1 | Matcap1 | Reps1 | Ube2d1 | Tesk1 | Pdc6i | Dst |
| 3.11 | Wbp11 | Zzef1 | Chd4 | Slc38a10 | Retreg1127 | Carm1 | Hnrnpl | Il6st |
| 3.12 | Aatf | Gtf3c1 | Lrif1 | Rdx | Phykpl | Lars | Spire1 | Nsd1 |
| 3.13 | Hdgfl2 | Rpl34 | Klf10 | Hoxb9 | | | | |

Assembly 2

| | | | | | | | | |
|---|---|---|---|---|---|---|---|---|
| 3.14 | Psmd2822 | Carm1 | Spire1 | Hdgfl2 | Gba2 | Tdg | Pi4kb | Sorbs1 |
| 3.15 | Lmo7 | Pdc6i | Slc25a45 | Rpl34 | Phldb2 | Snap47 | Zzef1 | Map1a |
| 3.16 | Med1 | Fcsk | Prxl2a | Gtf3c1 | Map7d1 | Cryzl1 | Raly | Ogfr |
| 3.17 | Slc35c1 | Dst | Med12 | Aldh3a2 | Lrif1 | Il6st | Ndc1 | Gtf2i |
| 3.18 | Get3 | Kcnv1 | Reps1 | Fads1 | Tbdg | Rdx | Hnrnpl | Adat1 |
| 3.19 | Lars | Dpagt1 | Rad50 | Ndufs6 | Sars | Zfp984 | Srebf1 | Zbtb40 |
| 3.20 | Retreg1127 | Srebf2 | Tet3 | Pop1 | Tesk1 | Hacd2 | Synj1 | Senp5 |
| 3.21 | Arhgap12 | Myo15 | Cplane1 | Cpne1 | N4bp2l2 | Cand1 | Tango6 | Nrbp2 |
| 3.22 | Trio | Ube2d1 | Irgq | Rpl18 | Ppp1r7 | Kif14 | Specc1 | Ankrd13a |
| 3.23 | Obscn | Eng | Matcap1 | Aatf | Psmd2755 | Ganab$_{733}$ | Ganab$_{394}$ | Paip1 |
| 3.24 | Hacd1 | Pdia5 | Wbp11 | Nsd1 | Btg1 | Agtpbp1 | | |
| 3.25 | Large1 | Slc38a10 | Eif4e2 | Phykpl | Ndrg1 | Skp2 | Retreg1288 | Klf10 |
| 3.26 | Kmt2b | Adpgk | Abcb8 | Hoxb9 | Ghr | Chd4 | | |

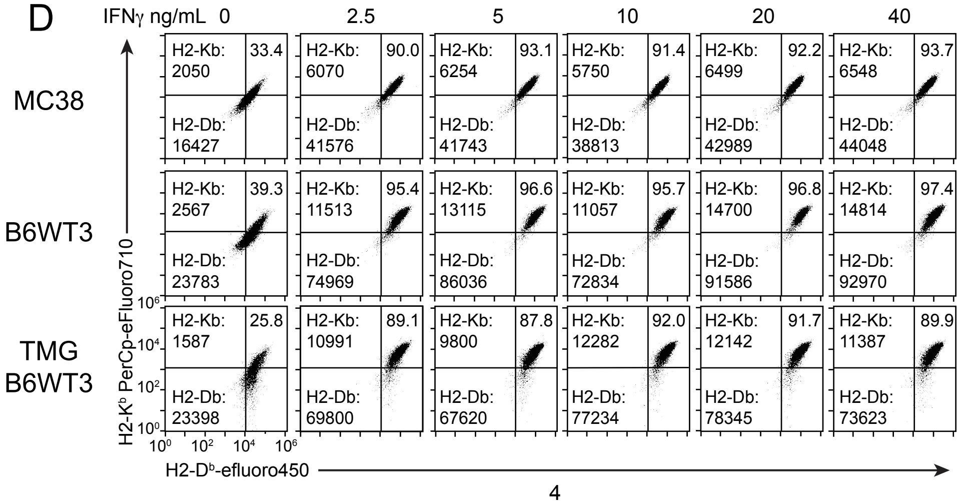

**Supplemental Figure 3. Isolation of additional anti-neoantigen TCRs in a second experiment.** (A) Tumors were implanted as in Figure 1 and $dex^{Rpl18-}dex^{ADPGK-}$ CD8 cells were single cell sorted (B; green area represents the sort gate). Jurkat cells were transduced with TCRs from this sort and puromycin-selected to increase TCR expression (C). Among the Jurkat lines selected for reaction against TMG-encoding APCs were those expressing TCRs isolated more than once. Shown in (D) are the CDR3 amino acid sequences and the number of times they were detected by Sanger sequencing of cloned TCRβ chains. Clones with low quality and uninterpretable CDR3 sequences were classified as "No ID". (E) TMG configurations for the second screening experiment. (F) Jurkat quintets were reacted against the Assembly 1 TMG array used in the first screening campaign (Library 1, Assembly 1; Supplemental Figure 2B) and Assembly 1 from the new library (Assembly 1, Library 2; panel E). Shown are the percentages of the quintet cultures expressing CD69. (G) Reactivity of quintet 19 against empty vector (EV), $Zc3h7b^-$ TMG 3.4 and $Zc3h7b^+$ TMG3.8-transduce B6WT3 cells. The single line expressing TCR J18 from mouse 7 (m7_J18) was the only reactive TCR (H). This TCR reacted against $Zc3h7b^+$ TMGs 4.8 and 4.23 but not $Zc3h7b^-$ TMGs 3.4 and 4.22 (I).

**Supplemental Figure 3. Isolation of additional anti-neoantigen TCRs in a second experiment.**

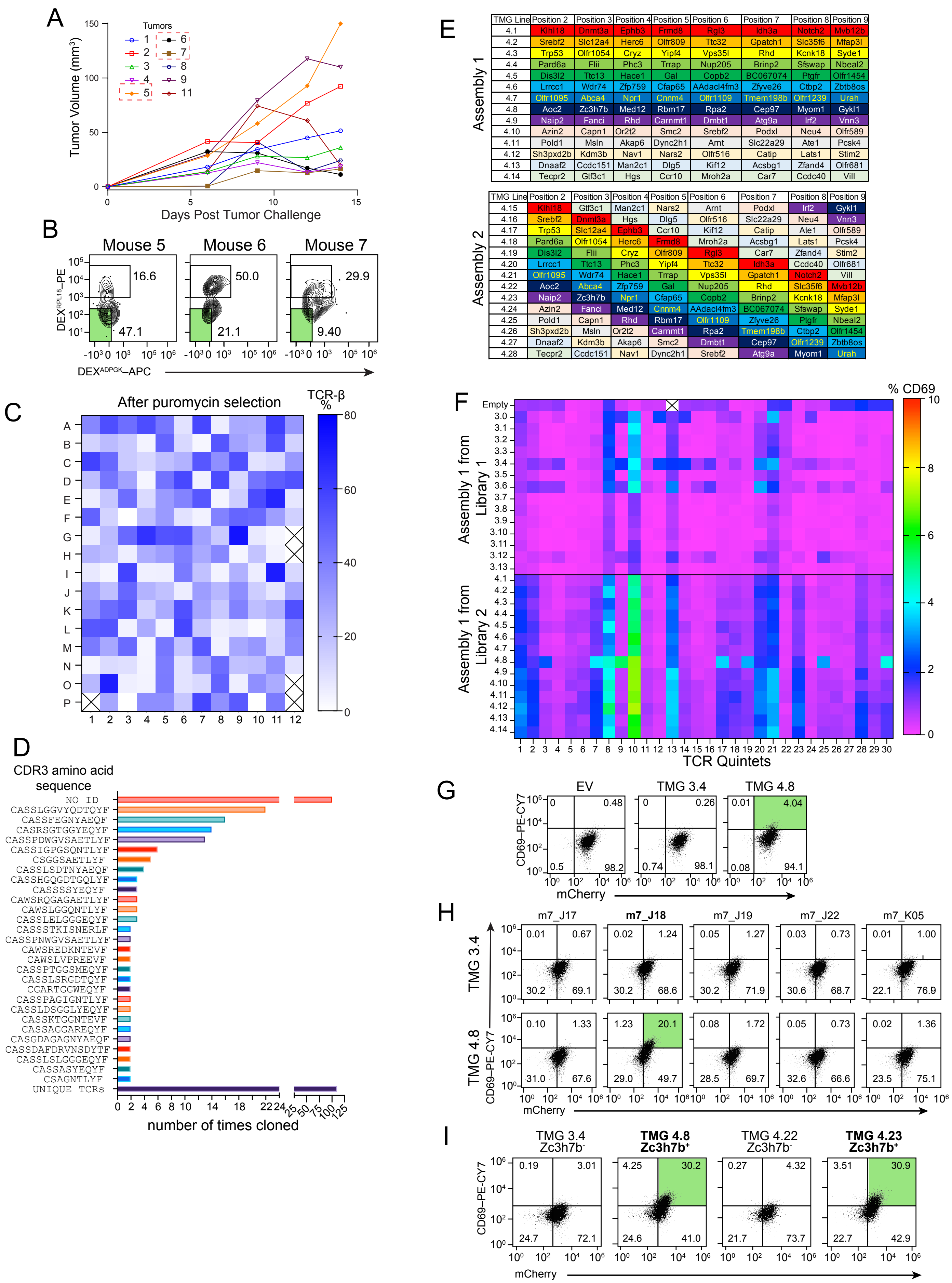


Assembly 1

| TMG Line | Position 2 | Position 3 | Position 4 | Position 5 | Position 6 | Position 7 | Position 8 | Position 9 |
|---|---|---|---|---|---|---|---|---|
| 4.1 | Klhl18 | Dnmt3a | Ephb3 | Frmd8 | Rgl3 | Idh3a | Notch2 | Mvb12b |
| 4.2 | Srebf2 | Slc12a4 | Herc6 | Olfr809 | Ttc32 | Gpatch1 | Slc35f6 | Mfap3l |
| 4.3 | Trp53 | Olfr1054 | Cryz | Yipf4 | Vps35l | Rhd | Kcnk18 | Syde1 |
| 4.4 | Pard6a | Flii | Phc3 | Trrap | Nup205 | Brinp2 | Sfswap | Nbeal2 |
| 4.5 | Dis3l2 | Ttc13 | Hace1 | Gal | Copb2 | BC067074 | Ptgfr | Olfr1454 |
| 4.6 | Lrrcc1 | Wdr74 | Zfp759 | Cfap65 | AAdacl4fm3 | Zfyve26 | Ctbp2 | Zbtb8os |
| 4.7 | Olfr1095 | Abca4 | Npr1 | Cnnm4 | Olfr1109 | Tmem198b | Olfr1239 | Urah |
| 4.8 | Aoc2 | Zc3h7b | Med12 | Rbm17 | Rpa2 | Cep97 | Myom1 | Gykl1 |
| 4.9 | Naip2 | Fanci | Rhd | Carnmt1 | Dmbt1 | Atg9a | Irf2 | Vnn3 |
| 4.10 | Azin2 | Capn1 | Or2t2 | Smc2 | Srebf2 | Podxl | Neu4 | Olfr589 |
| 4.11 | Pold1 | Msln | Akap6 | Dync2h1 | Arnt | Slc22a29 | Ate1 | Pcsk4 |
| 4.12 | Sh3pxd2b | Kdm3b | Nav1 | Nars2 | Olfr516 | Catip | Lats1 | Stim2 |
| 4.13 | Dnaaf2 | Ccdc151 | Man2c1 | Dlg5 | Kif12 | Acsbg1 | Zfand4 | Olfr681 |
| 4.14 | Tecpr2 | Gtf3c1 | Hgs | Ccr10 | Mroh2a | Car7 | Ccdc40 | Vill |

Assembly 2

| TMG Line | Position 2 | Position 3 | Position 4 | Position 5 | Position 6 | Position 7 | Position 8 | Position 9 |
|---|---|---|---|---|---|---|---|---|
| 4.15 | Klhl18 | Gtf3c1 | Man2c1 | Nars2 | Arnt | Podxl | Irf2 | Gykl1 |
| 4.16 | Srebf2 | Dnmt3a | Hgs | Dlg5 | Olfr516 | Slc22a29 | Neu4 | Vnn3 |
| 4.17 | Trp53 | Slc12a4 | Ephb3 | Ccr10 | Kif12 | Catip | Ate1 | Olfr589 |
| 4.18 | Pard6a | Olfr1054 | Herc6 | Frmd8 | Mroh2a | Acsbg1 | Lats1 | Pcsk4 |
| 4.19 | Dis3l2 | Flii | Cryz | Olfr809 | Rgl3 | Car7 | Zfand4 | Stim2 |
| 4.20 | Lrrcc1 | Ttc13 | Phc3 | Yipf4 | Ttc32 | Idh3a | Ccdc40 | Olfr681 |
| 4.21 | Olfr1095 | Wdr74 | Hace1 | Trrap | Vps35l | Gpatch1 | Notch2 | Vill |
| 4.22 | Aoc2 | Abca4 | Zfp759 | Gal | Nup205 | Rhd | Slc35f6 | Mvb12b |
| 4.23 | Naip2 | Zc3h7b | Npr1 | Cfap65 | Copb2 | Brinp2 | Kcnk18 | Mfap3l |
| 4.24 | Azin2 | Fanci | Med12 | Cnnm4 | AAdacl4fm3 | BC067074 | Sfswap | Syde1 |
| 4.25 | Pold1 | Capn1 | Rhd | Rbm17 | Olfr1109 | Zfyve26 | Ptgfr | Nbeal2 |
| 4.26 | Sh3pxd2b | Msln | Or2t2 | Carnmt1 | Rpa2 | Tmem198b | Ctbp2 | Olfr1454 |
| 4.27 | Dnaaf2 | Kdm3b | Akap6 | Smc2 | Dmbt1 | Cep97 | Olfr1239 | Zbtb8os |
| 4.28 | Tecpr2 | Ccdc151 | Nav1 | Dync2h1 | Srebf2 | Atg9a | Myom1 | Urah |

**Supplemental Figure 4. Molecular deconvolution of TCRs.** Plasmid isolated from the bulk bacterial cultures transformed with the anti-Rpl18 TCR H03 Gibson reaction was re-transduced into bacteria. Single colonies were picked, expanded and subject to plasmid isolation. Jurkat lines were created from these colony-derived plasmids (A) with the best TCR expression in those derived from picks 3, 6, 7 and 8 (in yellow). DNA sequencing was successful from picks 3 to 8, revealing 2 TRAV-TRAC junctions from two independent TCR$\alpha$ rearrangements. Jurkat lines from picks 4, 6, 7 and 8 were reacted against the Rpl18$^{mut}$ TMG 3.4 and negative control TMGs. Picks 6 and 8 expressing the common AASASSGSWQLIFGSGTQLTVMPD junction were reactive. A parallel process was used to deconvolute the anti-PSMD2 TCR G13. TCR expression from Jurkat lines derived from 8 colony picks is shown in (D). Sequencing revealed use of two different TCR$\alpha$ chains (E). Jurkat cells expressing pick 8, which encodes TRAV 12D-3, was reactive (F) when cultured against PSMD2$^{+}$ TMG APC 3.23 but was not reactive against empty vector and PSMD2$^{-}$ TMG3.4-expressing B6WT3 cells.

## Supplemental Figure 4. Molecular deconvolution of TCRs.

A

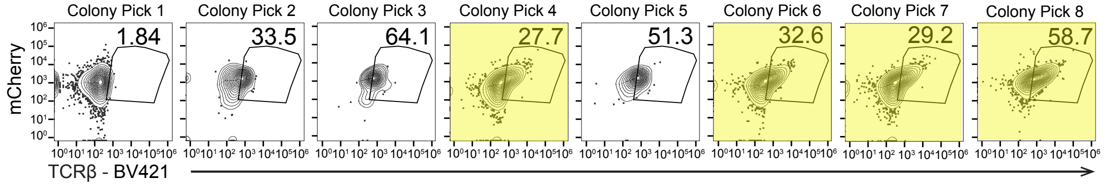


B

| COLONY PICK | TRAV | TRAV-TRAC JUNCTION | TRBV | TRBV-TRBC JUNCTION | TESTED |
|---|---|---|---|---|---|
| 1 | FAILED SEQUENCE | | | | NO |
| 2 | FAILED SEQUENCE | | | | NO |
| 3 | TRAV 14D-1 | TASATGSFNKLTFGAGTRLAVCPY | TRBV20 | TPQGSGNTLYFGEGSRLIVVE | NO |
| 4 | TRAV 14D-1 | TASATGSFNKLTFGAGTRLAVCPY | TRBV20 | TPQGSGNTLYFGEGSRLIVVE | YES |
| 5 | TRAV 14D-1 | TASATGSFNKLTFGAGTRLAVCPY | TRBV20 | TPQGSGNTLYFGEGSRLIVVE | NO |
| 6 | TRAV 14D-1 | AASASSGSWQLIFGSGTQLTVMPD | TRBV20 | TPQGSGNTLYFGEGSRLIVVE | YES |
| 7 | TRAV 14D-1 | TASATGSFNKLTFGAGTRLAVCPY | TRBV20 | TPQGSGNTLYFGEGSRLIVVE | YES |
| 8 | TRAV 14D-1 | AASASSGSWQLIFGSGTQLTVMPD | TRBV20 | TPQGSGNTLYFGEGSRLIVVE | YES |

C

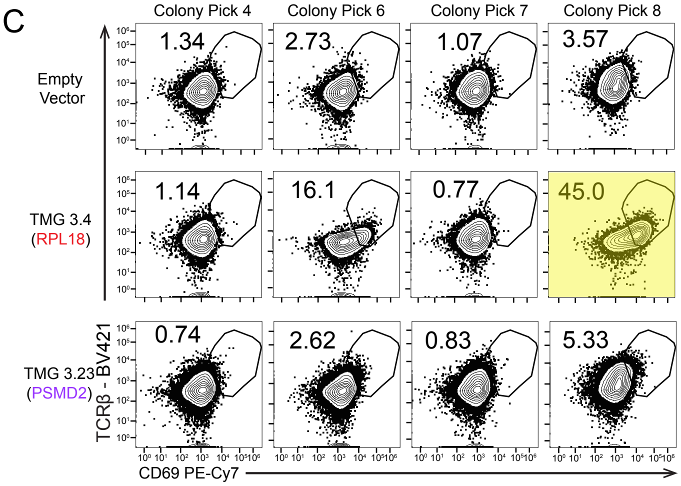


D

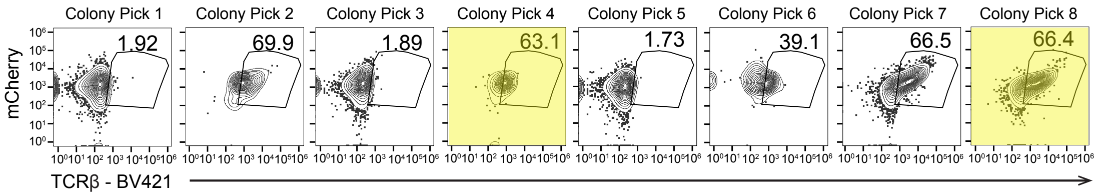


E

| COLONY PICK | TRAV | TRAJ-TRAC JUNCTION | TRBV | TRBJ-TRBC JUNCTION | TESTED |
|---|---|---|---|---|---|
| 1 | FAILED SEQUENCE | | | | NO |
| 2 | TRAV 3-1 | AKPGGYKVVFGSGTRLLVSP | TRBV2 | QDWGSQDTQYFGPGTRLLVLE | NO |
| 3 | FAILED SEQUENCE | | | | NO |
| 4 | TRAV 3-1 | AKPGGYKVVFGSGTRLLVSP | TRBV2 | QDWGSQDTQYFGPGTRLLVLE | YES |
| 5 | FAILED SEQUENCE | | | | NO |
| 6 | TRAV 3-1 | AKPGGYKVVFGSGTRLLVSP | TRBV2 | QDWGSQDTQYFGPGTRLLVLE | NO |
| 7 | FAILED SEQUENCE | | | | NO |
| 8 | TRAV 12D-3 | GNQGKLIFGQGTKLSIKP | TRBV2 | QDWGSQDTQYFGPGTRLLVLE | YES |

F

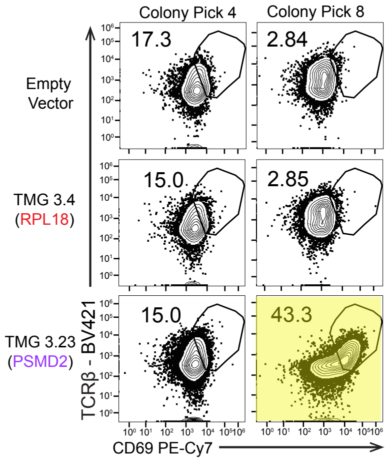

**Supplemental Figure 5. Characterization of autoreactive TCRs isolated from tumor-infiltrating CD8 cells.** Molecular deconvolution was performed on the positive Jurkat lines from quintets 9, 25, 27, 28 and 29 which resulted in at least 1 auto-reactive TCR from each quintet. Their reactivities to wild type parental, $K^{b-/-}$ and $K^{b-/-}D^{b-/-}$ B6WT3 cells and MC38 cells are shown in (A) and graphically in (B). The labels depict the tumor from which the TCR was isolated, the TCR name designation, and the originating quintet from which it was isolated. Negative controls for self-reactivity (bottom two rows) are deconvoluted TCRs against PSMD2 (clone G13) which reacts only against MC38 cells, and the anti-OVA TCR M19 that does not react against any APC.

**Supplemental Figure 5. Characterization of autoreactive TCRs isolated from tumor-infiltrating CD8 cells.**

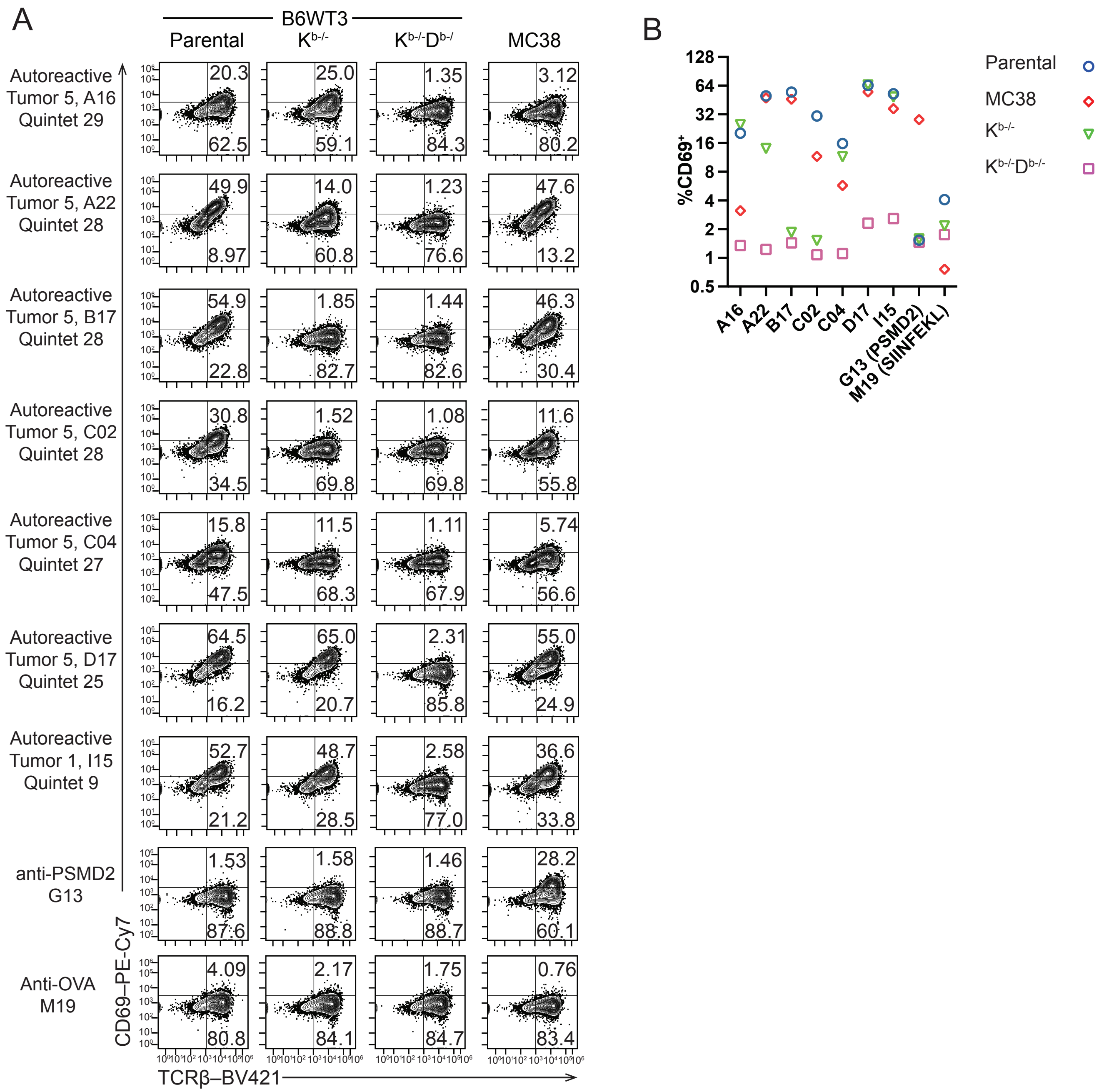

**Supplemental Figure 6. Creating and characterizing B16-TMG cells.** (A) A minigene construct (A) that expresses immunogenic epitopes from gp100 (EGSRNQDWL), H60 (LTFNYRNL), TRP1 (TAPDNLGYM), TRP2 (SVYDFFVWL) and OVA (SIINFEKL) along with eGFP was cloned into MSCV2.2 and retrovirus was used to infect B16F10 cells. Different subclones were created and tested for their ability to be killed by in vitro activated OT-1 TCR transgenic (Tg) T cells (B). Growth of B16-TMG and parental B16 cells were similar in B6 mice (C). B16-TMG or B16 parental cells were implanted intradermally in B6 mice. At day +20, tumors were harvested, and TIL were analyzed by flow cytometry. Both tumors were infiltrated with T cells (D, top row). $Tet^{H60+}$ and $dex^{OVA+}$ $CD8^{+}$ cells were only seen in B16-TMG tumors (D, second and third rows). B16-TMG cells were implanted intradermally in B6 mice, followed by infusion of activated polyclonal T cells or OT-1 TCR Tg cells and tumor diameters were measured (E). The AUC for the tumor growth in each mouse was calculated and the groups compared via the Mann-Whitney rank sum test.

**Supplemental Figure 6. Creating and characterizing B16-TMG cells.**

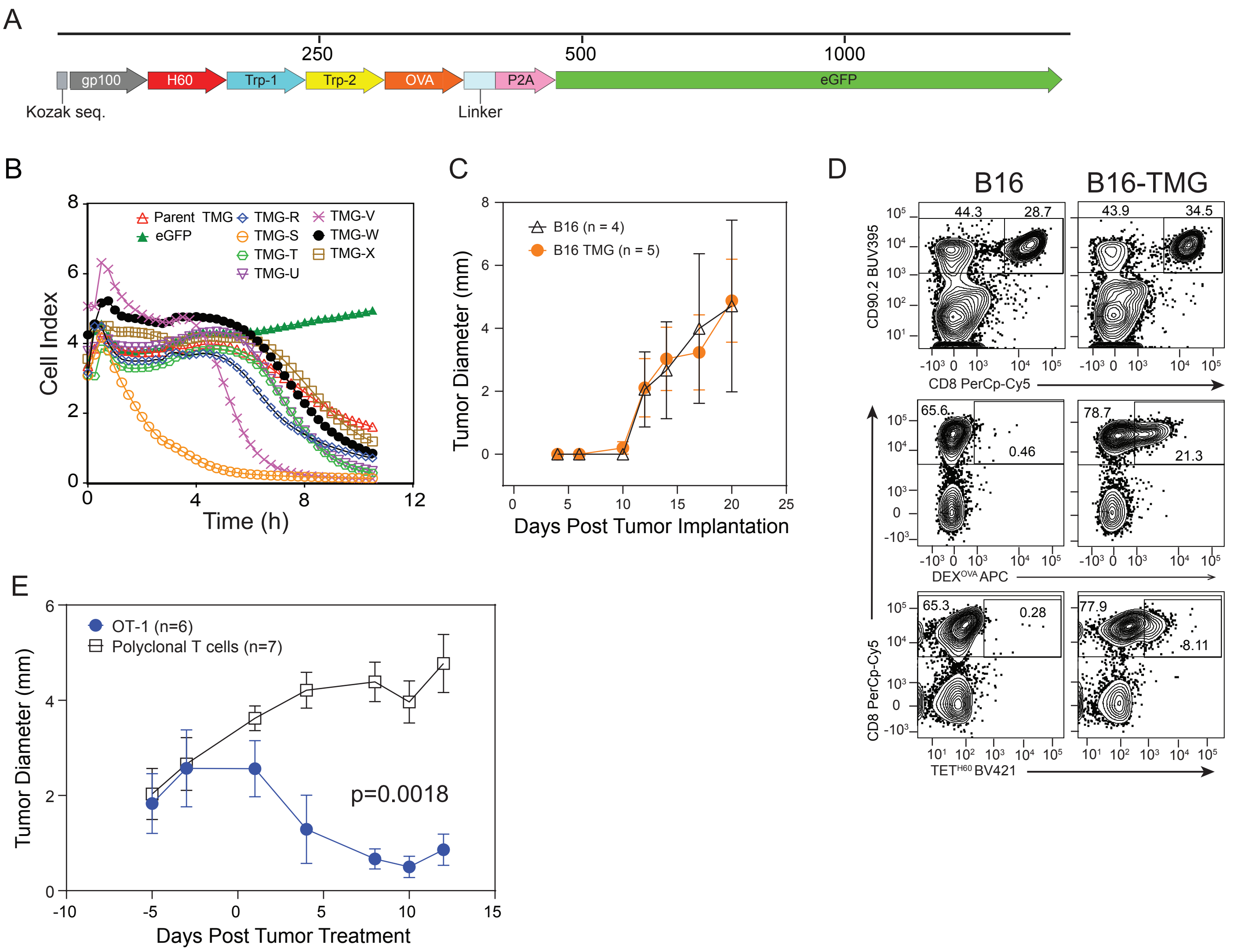

**Supplemental Figure 7. Titering of TCR-encoding retrovirus.** 4G4R cells in log-phase growth were transduced with different concentrations of retrovirus and TCRβ expression was quantitated by flow cytometry (A). These data (B) were used to calculate the number of infectious functional units (IFU) per microliter of supernatant determined when virus concentration versus percentage transduced were in a linear relationship as outlined in the red boxes in (B) and (C).

**Supplemental Figure 7. Titering of TCR-encoding retrovirus.**

A

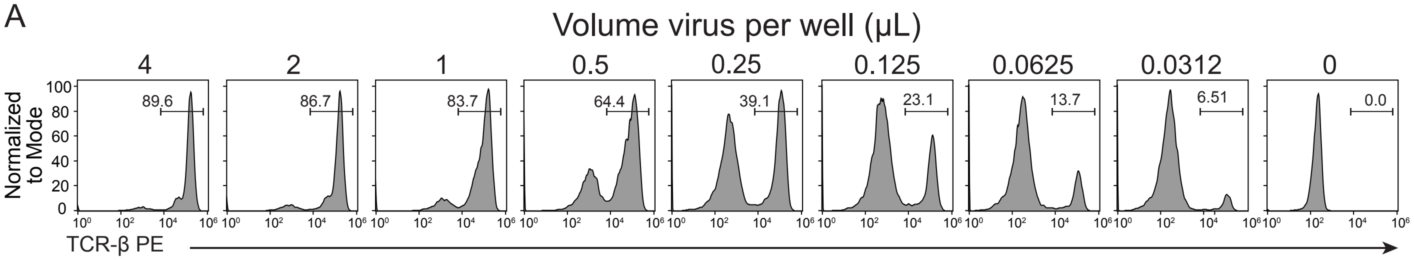


B

| μL virus/well | 4 | 2 | 1 | 0.5 | 0.25 | 0.125 | 0.0625 | 0.03125 |
|---|---|---|---|---|---|---|---|---|
| % transduced | 89.6 ±0.75 | 86.2 ±1.02 | 84.4 ±0.70 | 63.6 ±2.1 | 38.3 ±2.46 | 22.4 ±0.87 | 13.7 ±0.35 | 6.4 ±0.46 |
| IFU/well | 179266.67 ±1501.11 | 172333.33 ±2023.20 | 168866.67 ±1404.76 | 127133.3 ±4158.52 | 76533.33 ±5031.90 | 44733.33 ±1747.38 | 27466.67 ±702.38 | 12800.00 ±929.73 |
| IFU/μL | 44816.67 ±375.28 | 86166.67 ±1011.6 | 16886.67 ±1404.76 | 254266.67 ±8317.05 | 306133.33 ±19666.55 | 357866.33 ±13979.03 | 439466.67 ±11238.03 | 409600 ±29751.4 |

IFU/well = (cell/well) x % transduced

IFU/μL= (IFU/well) / (μL virus/well)

Avg. IFU/μL= average of linear portion of the curve

C

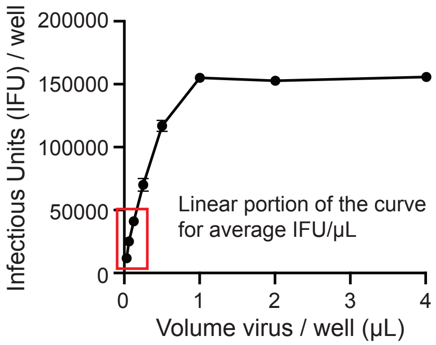

**Supplemental Figure 8. Data in support of Figure 4.** Survival of mice in experiment depicted in Figure 4 (A). Representative staining of T cells extracted from mice at sacrifice from spleen (B) and tumor (C).

**Supplemental Figure 8. Data in support of Figure 4.**

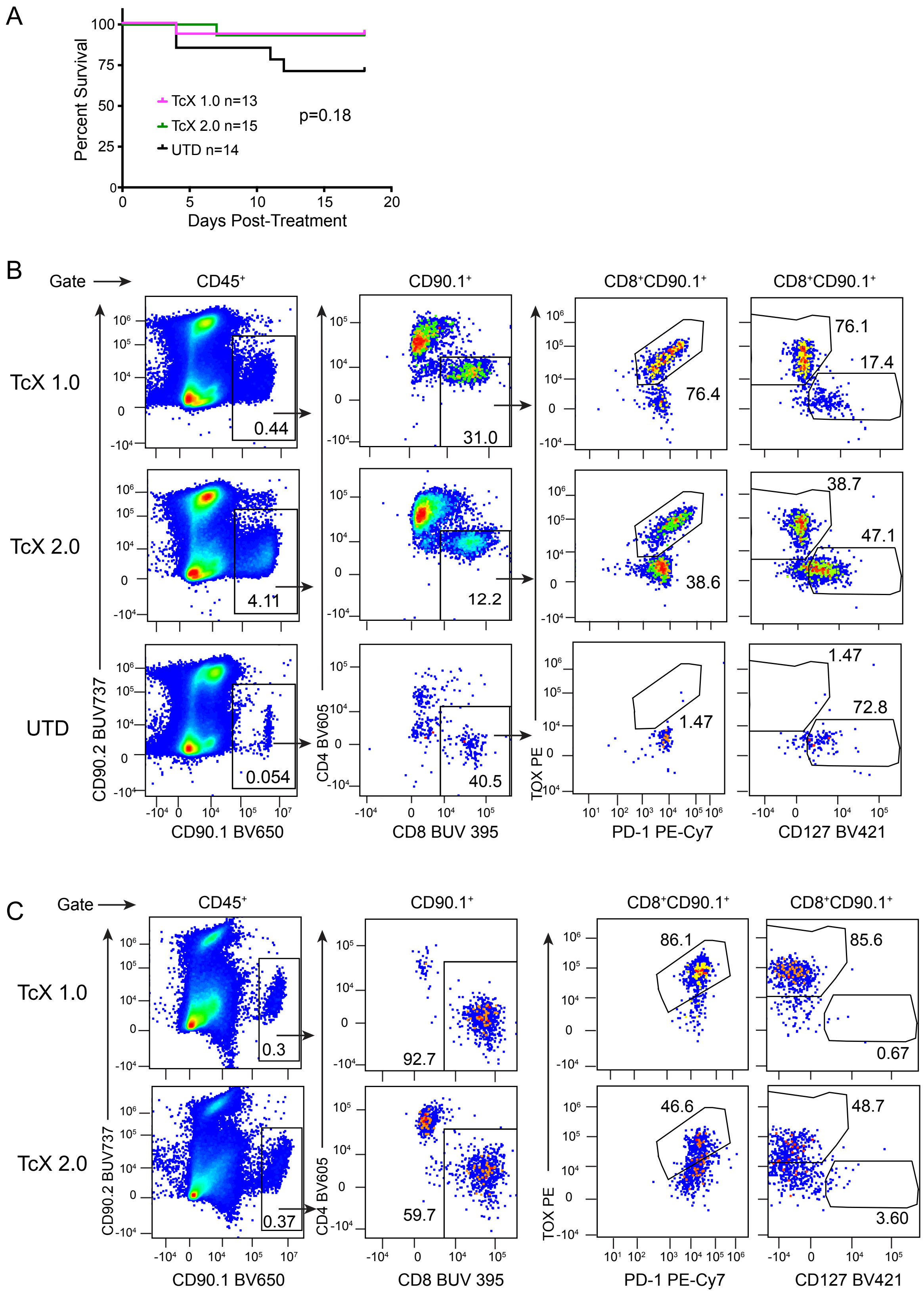

**Supplemental Figure 9. Anti-Zc3h7b TCRs recognize mutant but not wild-type Zc3H7b-encoding minigenes.** Jurkat lines expressing anti-mutant Zc3h7b TCRs were reacted against B6WT3 cells expressing TMGs including the mutant Zc3H7b epitope (TMGs 4.8 and 4.23) and their counterparts modified to express wild type (WT) Zc3H7b. Shown is CD69 versus mCherry expression with all Jurkat lines only reacting with TMGs encoding mutant Zc3H7b.

**Supplemental Figure 9. Anti-Zc3h7b TCRs recognize mutant but not wild-type Zc3H7b-encoding minigenes.**

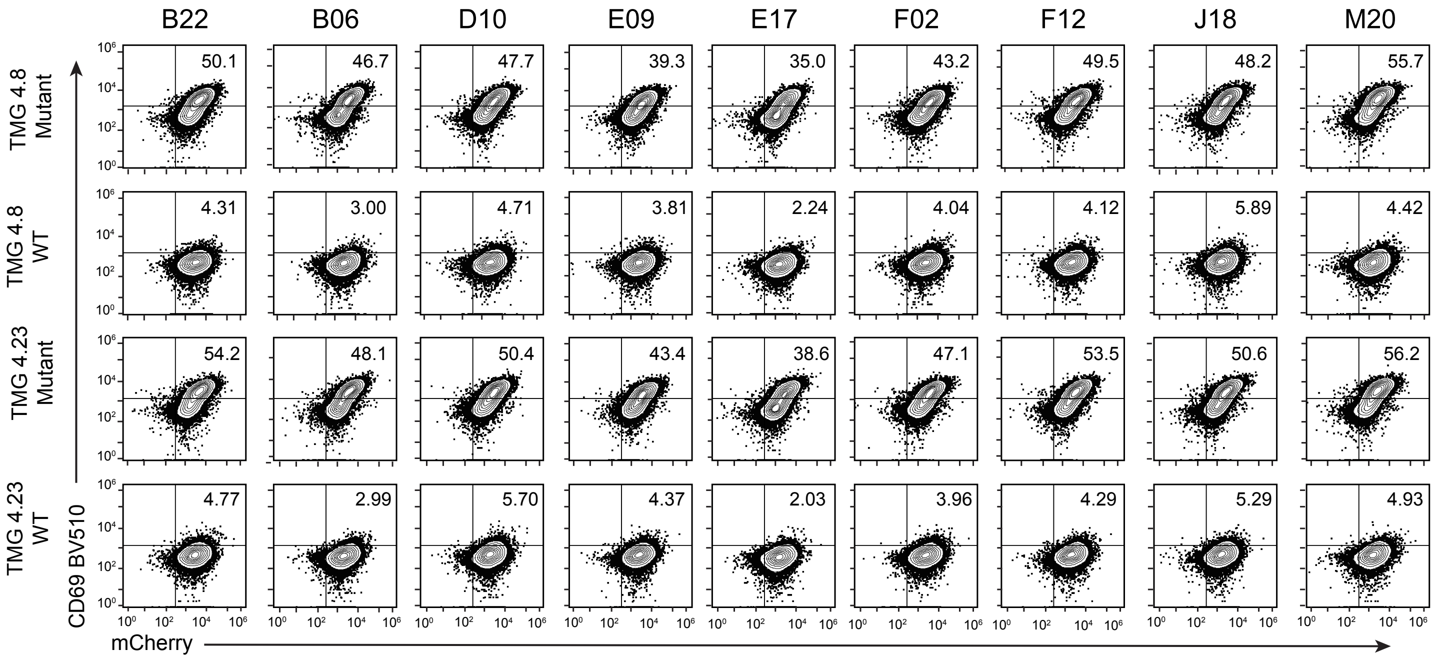

**Supplemental Figure 10. Killing of targets by anti-ADPGK TCRs.** Primary T cell products were created using virus encoding anti-ADPGK TCRs A10 and TCR30, the anti-Rpl18 TCR A09, or the anti-OVA TCR M19 using the TCX2.0 process with CRISPR-editing of the endogenous TCRs. Shown in (A) are TCRβ expression and $dex^{Rpl18}$ binding to these products along with T cells that underwent the TCX2.0 process without TCR transduction, with or without CRISPR TCR-editing (first two columns). A10, A09 and TCR30 products killed MC38 cells relative to M19 T cells (B). The same products were tested for their ability to kill B6WT3 cells expressing TMG 4.23 which expresses OVA but not Rpl18 or ADPGK (C), TMG 3.4 which expresses Rpl18 and OVA but not ADPGK (D), TMG3.6 which expresses ADPGK and OVA but not Rpl18 (E) and against parental unmodified B6WT3 cells (F). P values were determined using a one-way ANOVA with a Dunnett's multiple comparisons test. Data are representative of at least two similar experiments with similar results.

**Supplemental Figure 10. Killing of targets by anti-ADPGK TCRs.**

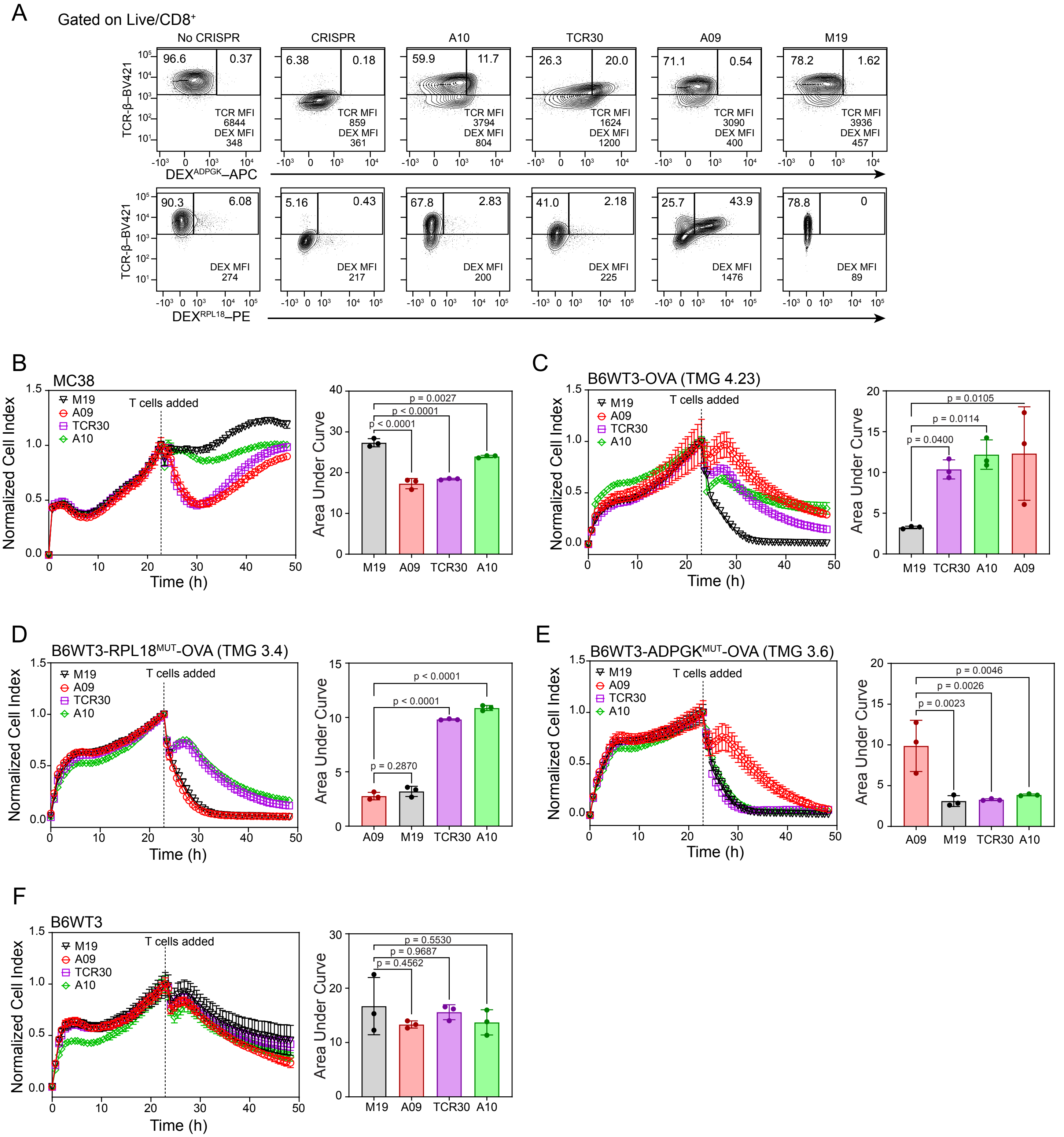

**Supplemental Figure 11. Data in support of Figure 7.** Tumor volumes from individual mice from the experiment depicted in Figure 7B-C (A). Representative flow cytometry of splenocytes (B) and TIL (C).

**Supplemental Figure 11. Data in support of Figure 7.**

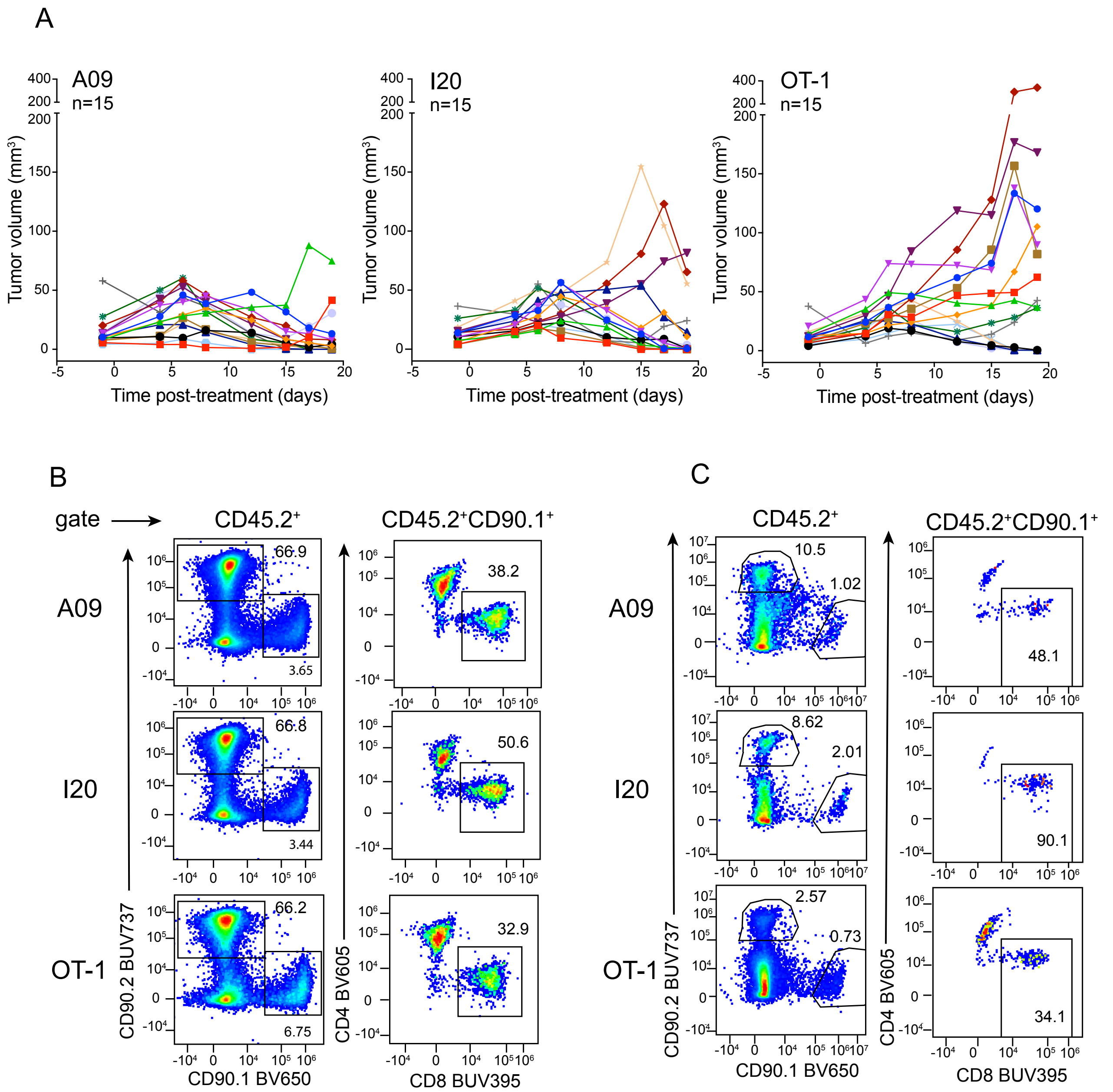

Supplement: Supplemental data [file jciinsight-11-203622-s281.pdf]
